# Methodologies for Evaluating the Usability of Rehabilitation Technologies Aimed at Supporting Shared Decision-Making: Scoping Review

**DOI:** 10.2196/41359

**Published:** 2023-08-15

**Authors:** Rehab Alhasani, Nicole George, Dennis Radman, Claudine Auger, Sara Ahmed

**Affiliations:** 1 Department of Rehabilitation Sciences, College of Health and Rehabilitation Sciences, Princess Nourah bint Abdulrahman University Riyadh Saudi Arabia; 2 School of Physical and Occupation Therapy, Faculty of Medicine, McGill University Montreal, QC Canada; 3 Centre for Interdisciplinary Research in Rehabilitation of Greater Montreal Montreal, QC Canada; 4 Institut Universitaire sur la Réadaptation en Déficience Physique de Montréal, Centre Intégré Universitaire de Santé et de Services Sociaux du Centre-Sud-de-l’Île-de-Montreal Montreal, QC Canada; 5 School of Rehabilitation, Faculty of Medicine, University of Montreal Montreal, QC Canada; 6 Constance Lethbridge Rehabilitation Center, Centre Intégré Universitaire de Santé et de Services Sociaux du Centre-Ouest-de-l’Île-de-Montreal Montreal, QC Canada; 7 McGill University Health Center Research Institute, Centre for Health Outcomes Research Montreal, QC Canada

**Keywords:** usability, technology, rehabilitation, shared decision-making, mobile phone

## Abstract

**Background:**

The field of rehabilitation has seen a recent rise in technologies to support shared decision-making (SDM). Usability testing during the design process of SDM technologies is needed to optimize adoption and realize potential benefits. There is variability in how usability is defined and measured. Given the complexity of usability, a thorough examination of the methodologies used to measure usability to develop the SDM technologies used in rehabilitation care is needed.

**Objective:**

This scoping review aims to answer the following research questions: which methods and measures have been used to produce knowledge about the usability of rehabilitation technologies aimed at supporting SDM at the different phases of development and implementation? Which parameters of usability have been measured and reported?

**Methods:**

This review followed the Arksey and O’Malley framework. An electronic search was performed in the Ovid MEDLINE, Embase, CINAHL, and PsycINFO databases from January 2005 up to November 2020. In total, 2 independent reviewers screened all retrieved titles, abstracts, and full texts according to the inclusion criteria and extracted the data. The International Organization for Standardization framework was used to define the scope of usability (effectiveness, efficiency, and satisfaction). The characteristics of the studies were outlined in a descriptive summary. Findings were categorized based on usability parameters, technology interventions, and measures of usability.

**Results:**

A total of 38 articles were included. The most common SDM technologies were web-based aids (15/33, 46%). The usability of SDM technologies was assessed during development, preimplementation, or implementation, using 14 different methods. The most frequent methods were questionnaires (24/38, 63%) and semistructured interviews (16/38, 42%). Satisfaction (27/38, 71%) was the most common usability parameter mapped to types of SDM technologies and usability evaluation methods. User-centered design (9/15, 60%) was the most frequently used technology design framework.

**Conclusions:**

The results from this scoping review highlight the importance and the complexity of usability evaluation. Although various methods and measures were shown to be used to evaluate the usability of technologies to support SDM in rehabilitation, very few evaluations used in the included studies were found to adequately span the selected usability domains. This review identified gaps in usability evaluation, as most studies (24/38, 63%) relied solely on questionnaires rather than multiple methods, and most questionnaires simply focused on the usability parameter of satisfaction. The consideration of end users (such as patients and clinicians) is of particular importance for the development of technologies to support SDM, as the process of SDM itself aims to improve patient-centered care and integrate both patient and clinician voices into their rehabilitation care.

## Introduction

### Background

Shared decision-making (SDM), the collaborative process involving active participation from both patients and providers in health care treatment decisions, reflects an important paradigm shift in medicine toward patient-centered care [1,2]. SDM facilitates information exchange and discussion of treatment options that involve the best scientific evidence and consider patient preferences [3,4]. The readiness for using SDM may be enhanced through its accessibility to individuals with limited health literacy or those with disabilities [5]. In the context of rehabilitation, SDM typically occurs during goal setting by selecting and agreeing upon behavioral objectives that patients, caregivers, and the rehabilitation team work together to achieve [6]. The development of mutual trust, 2-way communication, and sharing of power are conditions that influence patients’ capacity and confidence to participate in SDM in musculoskeletal physiotherapy [7] and in the treatment of depression [8]. As a result, SDM assists patients in making individualized care decisions, and health care providers can feel confident in the presented and prescribed options [3,4]. SDM is important to increase satisfaction with care among both patients and providers, may improve individuals’ quality of life and clinical outcomes, and fosters a better patient-provider relationship [9]. Furthermore, SDM encourages patient participation in their rehabilitation, supporting self-efficacy, empowerment, and ownership over the decisions [6].

Despite the listed benefits, it has been difficult to implement SDM in clinical practice because of barriers such as time constraints, accessibility to information and effective SDM tools, and limited technical and organizational resources [3]. It has been reported that only 10% of face-to-face clinical consultations involve SDM [10,11]. Advances in digital health technologies (eHealth) have resulted in tools that can bridge this SDM gap by allowing increased access to shared information and support for patient-provider communication [12]. Accessible, cost-effective, web-based decision-making is supported by use across various platforms such as the internet, tablets, or smartphone apps [13,14]. Such SDM technologies include patient decision aids that clarify options and values for personalized decision support, leading to reduced decisional conflict and increased participation in treatment choices that are consistent with the patient’s values [13]. Patient portals reflect another technology that can support SDM, providing patients with secure access to their health information profile and communication with their care provider [15-17].

Although studies have been conducted to introduce and investigate the acceptance of rehabilitation technologies, research into the usability of technology systems is limited [18,19]. A technology system in rehabilitation is defined as an environmental factor that incorporates aspects of the physical and social environments that may affect communicative participation [20]. Technology systems need to be evaluated in terms of their usability to maximize their acceptance and benefits. The International Organization for Standardization (ISO) 9241 defined usability as “the extent to which a product can be used by specified users to achieve specified goals with effectiveness, efficiency, and satisfaction in a specified context of use” [21]. Evaluation of usability is key to guiding the development of efficient and effective technologies that end users will readily adopt by providing information about how a user uses the technology system and the challenges they find while interacting with a system’s interface [22]. Different usability models have been proposed for evaluating software usability. Gupta et al [23] proposed a comprehensive hierarchal usability model with a detailed taxonomy, including 7 usability parameters: efficiency, effectiveness, satisfaction, memorability, security, universality, and productivity. Evaluating these usability parameters throughout the design process can allow for continuous improvement of ease of use and can predict the user’s acceptance or rejection of the product [24]. Therefore, including input from individuals who will use the technology (in the case of SDM technologies, clinicians, patients, and caregivers) through usability testing is a necessary component in designing relevant, understandable, and usable technologies.

### Objectives

The field of rehabilitation science is defined as a multidimensional person-centered process targeting body functions, activities and participation, and the interaction with the environment aiming at optimizing functioning among persons with health conditions experiencing disability [25]. It has seen a recent rise in the development and implementation of technologies aimed at supporting SDM between clinicians, patients, and their caregivers [26]. However, it is unclear how user input or usability testing is integrated into the design process of these rehabilitation health technologies, including how usability is conceptualized, what measures are used, and at what stage of design usability is evaluated. To date, few studies, and no systematic or scoping reviews that we are aware of, have addressed how usability is measured among rehabilitation technologies supporting SDM. Given the complexity of usability, a thorough examination of the methodologies used to measure usability in this context is required to comprehensively map what has been done and inform future research efforts. A greater understanding of how the parameters of usability are measured will guide future usability testing to inform further development of SDM technologies designed to enhance patient-centered care in rehabilitation. Therefore, this scoping review was conducted to provide knowledge about the methods and measures used to determine the usability of rehabilitation technologies aimed at supporting SDM at different phases of technology development and implementation.

## Methods

**Figure 1:**
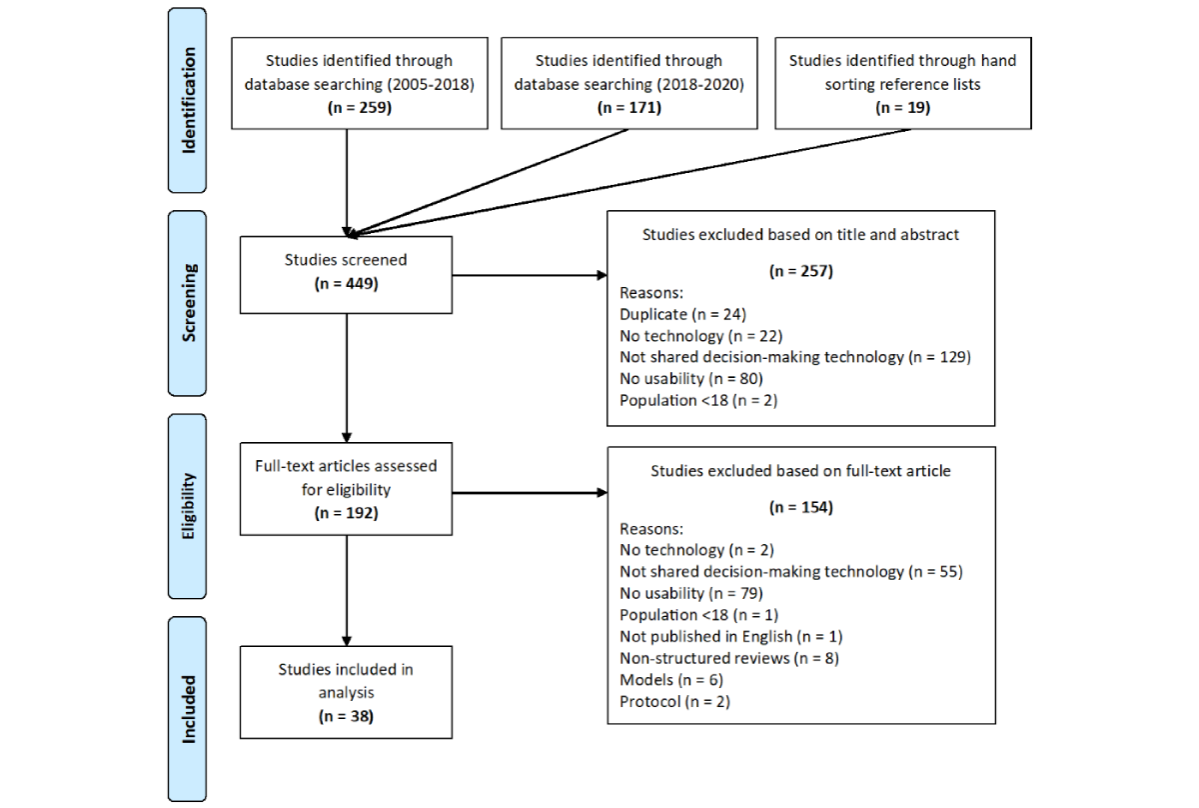
PRISMA-ScR (Preferred Reporting Items for Systematic Reviews and Meta-Analyses extension for Scoping Reviews) flow diagram.

This scoping review followed the methodology described by Arksey and O’Malley [27] and was reported according to the PRISMA-ScR (Preferred Reporting Items for Systematic Reviews and Meta-Analyses extension for Scoping Reviews) guidelines [28] (Figure 1).

### Identifying the Research Questions

This scoping review aimed to answer the following research questions: (1) which methods and measures have been used to produce knowledge about the usability of rehabilitation technologies aimed at supporting SDM at the different phases of development and implementation? (2) Which parameters of usability have been measured and reported in studies focusing on rehabilitation technologies aimed at supporting SDM?

### Eligibility Criteria

The eligibility criteria for this scoping review are outlined in Textbox 1.

**Inclusion and exclusion criteria.**

**Inclusion criteria**

- Articles published in peer-reviewed journals, including quantitative (randomized controlled trials or nonrandomized controlled trials), qualitative, and mixed methods studies
- Articles including different groups of people, such as health care practitioners and individuals seeking rehabilitation services (ie, patients and their caregivers) or case managers
- Articles that focused on the usability of technology in making decisions
- Articles reporting a clear objective to evaluate the usability of shared decision-making (SDM) technologies in rehabilitation

**Exclusion criteria**

- Nonstructured reviews, protocols, descriptive reviews, nonhuman studies, and gray literature
- Articles not focusing on or measuring the usability of technologies in SDM and groups not related to the health care sector (ie, students)

### Search Strategy

The search strategy was developed in collaboration with a health science librarian. As health system issues often change with models of care delivery, the economic climate, and the environment [29], we decided to narrow the scope of the search (2005 to 2020). The following electronic databases were searched in both English and French: Ovid MEDLINE, Embase, CINAHL, and PsycINFO. A combination of Medical Subject Heading terms, subject headings, and keywords was used and covered five concepts: (1) *usability* OR user* friendl* OR eas* to use OR useful* OR user* perspective* OR patient* perspective* OR client* perspective* OR user* experience* AND (2) *rehabilitation* OR *telerehabilitation* OR *tele rehabilitation* OR *disabled* OR disabilit* OR physical limitation* OR mental limitation* OR psycho* limitation* OR adaptation* OR *mobility* OR occupational therap* OR physiotherap* OR physical therap* OR speech languag* pathol* OR speech therap* OR language therap* OR communication disorder* AND (3) think* aloud OR focus group* OR interview* OR Wizard* OR Empathy map* OR Persona* OR Questionnaire* OR instrument* OR scale* OR *tool* OR *tools* OR measurement* OR survey* OR *drama* OR deliberation* OR evaluation* OR assessment* OR video confrontation* OR photo voice* AND (4) technolog* OR gerontotechnolog* OR smart* OR intelligen* OR *ambient assisted living* OR *virtual reality* OR *virtual rehabilitation* OR *telemonitoring* OR *telehealth* OR *telemedicine* OR *telerehabilitation* OR *ehealth* OR *tele monitoring* OR *tele health* OR *tele medicine* OR *tele rehabilitation* OR e health or sensor* OR biosensor* OR mobile app* OR product* OR *internet* OR *web* OR computer* OR software* OR device* OR *self-help* OR wheelchair* OR wheelchair* OR communication aid* AND (5) *shared decision making* OR *Decision-Making* OR *patient-provider communication* OR *decision aid* OR *decision support*. This was followed by hand searches of the reference lists of the included studies (the search strategy for Ovid MEDLINE is presented in Multimedia Appendix 1).

### Study Selection

All identified studies were uploaded into EndNote X9.1 (Clarivate Analytics), and duplicates were removed. In total, 2 independent reviewers conducted the selection of abstracts starting with a pilot phase involving the examination of the first 10 titles and abstracts to screen and decide on retention of the abstract based on the inclusion criteria. Interrater agreements were assessed using the κ statistic [30]. Interrater agreement of <75% resulted in a clarification of the eligibility criteria and a revision if needed. The process was repeated twice between the reviewers until an agreement of 75% was reached, which is evidence of excellent agreement [30]. Finally, all eligible studies and those classified as unclear (ie, requiring further information to make a final decision regarding their retention) were independently reviewed as full-text articles. Disagreements at this stage were resolved through consensus. The PRISMA-ScR flow diagram [28] was used to guide the selection process.

### Data Extraction

In total, 2 reviewers independently extracted data from the included articles to avoid missing relevant information. The data extracted included information corresponding to study design, rehabilitation technology intervention used (ie, setting, content, and detail of the type of user interface), population studied (participant demographics and target conditions), characteristics of the measures, and the development stage.

### Data Synthesis

Descriptive statistics were used to describe the characteristics of the included studies, study design, characteristics of the study population, and geographical location. Findings were categorized based on study designs, parameters of usability, types of technologies, stage of development of the technology, and usability evaluation methodologies.

Types of SDM technologies and usability evaluations were mapped to parameters of usability based on a comprehensive hierarchal usability model presented by Gupta et al [23]. The usability parameters include efficiency, defined as “enables user to produce desired results with respect to investment of resources”; effectiveness, defined as “a measure of software product with which user can accomplish specified tasks and desired results with completeness and certainty”; satisfaction, defined as “a measure of responses, feelings of user when users are using the software i.e., freedom from discomfort, likeability”; memorability, defined as “the property of software product that enables the user to remember the elements and the functionality of the system product”; security, defined as “the degree to which risks and damages to people or other resources i.e. hardware and software can be avoided”; universality, defined as “the accommodation of different cultural backgrounds of diverse users with software product and practical utility of software product”; and productivity, defined as “the amount of useful output with the software product” [28] (Textbox 2).

The usability evaluation methodologies were mapped based on the framework by Jacobsen [31]. The categories of the usability evaluation methods included (1) empirical methods, based on users’ experience with the technology in a systematic way; (2) inspection methods, conducted by experts who examined usability-related aspects of a user interface without involving any users; and (3) inquiry methods, based on the information about users’ needs, likes, and understanding of the technology through interviews or focus groups, observation, and verbal or written questions [31].

**Usability parameters based on a comprehensive hierarchal usability model presented by Gupta et al [23].**

**Efficiency**

- Resources
- Time
- User effort
- Economic
- Cost

**Effectiveness**

- Task accomplishment
- Operability
- Extensibility
- Reusability
- Scalability

**Satisfaction**

- Likability
- Convenience
- Esthetics

**Memorability**

- Learnability
- Memorability of structure
- Comprehensibility
- Consistency of structure

**Security**

- Safety
- Error tolerance

**Universality**

- Approachability
- Utility
- Faithfulness
- Cultural universality

**Productivity**

- Useful user task output

### Consulting and Translating Knowledge

This scoping review is part of an initiative (*Réseau provincial de recherche en adaptation-réadaptation*–*RS6 Technologies de readaptation* [Quebec Rehabilitation Research Network]; [6]) to create an interactive directory of methodological tools for measures of the usability of rehabilitation technologies. Stakeholder consultations with members of the *Réseau provincial de recherche en adaptation-réadaptation*–RS6 group were held at the beginning of the process (requesting feedback to refine the research question for data extraction and synthesis), during the study (validating the data extraction and deciding on the best way to align the information with stakeholders’ needs), and when the final results were available (knowledge mobilization).

## Results

### Study Selection

A total of 430 studies were identified from electronic searches, and a total of 19 were identified through hand sorting reference lists. We excluded 57.2% (257/449) of the studies at the title and abstract stage, resulting in 192 full-text articles. Of these 192 studies, 154 (80.2%) were excluded at the full-text stage, resulting in 38 (19.8%) studies. The search strategy was updated in November 2020 and followed the PRISMA-ScR flowchart of the selection process. Reasons for exclusion of studies are provided in Figure 1. Interrater agreement reached ≥75%, which is evidence of excellent agreement. Disagreements were resolved through consensus.

### Characteristics of the Included Studies

The characteristics of the included studies are presented in Multimedia Appendix 2 [32-69]. Overall, the 38 included studies were published between 2008 and 2020 as peer-reviewed studies. Studies were published in the United States (17/38, 43%), Europe (14/38, 37%), Canada (5/38, 13%), and Asia (2/38, 5%). The study designs of the included studies were mixed methods (20/38, 53%), qualitative (12/38, 31%), and quantitative (6/38, 16%).

### Characteristics of the Included Participants

Multimedia Appendix 2 presents the characteristics of the included participants. The number of participants across all the included studies was 2138, with age ranging between 18 and 86 years. Participants of usability evaluations included patients (38/38, 100%); clinicians (32/38, 84%); caregivers or family (12/38, 32%); and others (6/38, 16%), including case managers, drug advisory committees, computer scientists, behavioral scientists, communication scientists, clinical administrators, service providers, and social service providers. The target end users of the developed SDM technologies were mainly patients and clinicians (24/38, 63%). The recruitment methods and settings varied across the included studies, including hospitals (24/38, 63%), the community (10/38, 26%), and universities (4/38, 11%).

### Usability Definitions and Parameters

Table 1 presents usability definitions and parameters provided by the authors across the included studies. Notably, only 50% (19/38) of the included studies provided an a priori definition of usability or listed parameters of usability. Usability parameters were categorized as effectiveness (9/38, 23%), efficiency (8/38, 21%), memorability (11/38, 29%), satisfaction (14/38, 37%), security (5/38, 13%), universality (4/38, 10%), and productivity (10/38, 26%) based on Gupta et al [23].

**Table 1:** Usability definitions and parameters.

Study	Definition of usability^a^	Usability parameters^a^	Gupta et al [23] framework
Bauerle Bass et al [34], 2018	User testing was completed to assess the extent to which the tool was understandable, how easily it could be navigated, and its relevance to patients taking HCV^b^+methadone.	Understandable Navigation Relevance	Memorability Memorability Productivity
Berry et al [35], 2015	Usability testing is the evaluation of information systems through testing by representative users, enabling evaluation of social acceptability, practicality, and usability of a technology.	Social acceptability Practicality Navigation Content comprehension Sociocultural appropriateness	Satisfaction Productivity Memorability Memorability Universality
Bogza et al [36], 2020	NR^c^	Acceptability Satisfaction	Satisfaction Satisfaction
Chrimes et al [39], 2014	Refers to commentary on the perceived effectiveness, efficiency, and ease of use, or lack thereof, of the ADAPT^d^ Toolkit.	Effectiveness Efficiency Ease of use	Effectiveness Efficiency Satisfaction
Cox et al [40], 2015	Usability describes the quality of a user’s experience with software or an IT considering their own needs, values, abilities, and limitations.	Quality of experience	Productivity
Cuypers et al [41], 2019	NR	Layout Language Content Amount Value clarification Summary	Effectiveness Memorability Memorability Memorability Effectiveness Memorability
Danial-Saad et al [43], 2016	Usability is defined by the ISO^e^ 9241 as the “extent to which a product can be used by specified users to achieve specified goals with effectiveness, efficiency and satisfaction in a specified context of use.”	Learnability Efficiency Memorability Errors Satisfaction Visibility Affordance User control Consistency User-friendliness	Memorability Efficiency Memorability Security Satisfaction Memorability Universality Productivity Memorability Satisfaction
De Vito Dabbs et al [42], 2009	The measure of the ease with which a system can be learned and used, including its safety, effectiveness, and efficiency.	Learnability Effectiveness Efficiency Errors Flexibility Memorability User satisfaction	Memorability Effectiveness Efficiency Security Satisfaction Memorability Satisfaction
Fleisher et al [44], 2008	Whether patients found the tools easy to use and navigate, as well as the readability and usefulness of the physician report. Usability protocol based on NCI^f^ guidelines.	Ease of use Readability Usefulness	Satisfaction Satisfaction Productivity
Fu et al [46], 2020	Usability is defined by the ISO 9241-11 as the extent to which a product can be used by a specific person in a specific context to achieve realistic goals of effectiveness, efficiency, and satisfaction.	Help and documentation Error prevention Esthetic and minimalist design Flexibility and efficiency of use Recognition rather than recall Match between app and the real world User control and freedom Consistency and standards Feedback and visibility Helps recover from errors	Productivity Security Satisfaction Efficiency Memorability Efficiency Universality Universality Effectiveness Productivity
Goud et al [47], 2008	NR	Ease of system use Information quality Interface quality	Satisfaction Memorability Efficiency
Grim et al [48], 2017	Usability refers to commentary. Understandability and usefulness are 2 major constructs when talking about usability. Understandability refers to the extent to which the descriptive texts and items are comprehensible. Usefulness refers to commentary on the extent to which the features in the decision aid are perceived as supporting decision-making processes on the perceived effectiveness, efficiency, and ease of use, or lack thereof, of the decision aid.	Understandability Usefulness	Memorability Productivity
Kallen et al [52], 2012	Usability was considered an incorporation of system effectiveness, efficiency, and user satisfaction. Usability was defined in the context of the assessment and review of tasks assigned to study participants.	System effectiveness Efficiency User satisfaction	Effectiveness Efficiency Satisfaction
Li et al [53], 2013	A usability issue was defined as (1) when a participant was not able to advance to the next step because of the decision aid design or a programming error or (2) when a participant was distracted by a particular design or content of the web tool.	Errors Design	Security Effectiveness
Rochette et al [55], 2008	The term “usability” is defined as the effectiveness, efficiency, and satisfaction with which users can achieve tasks in a particular environment. High usability means that a system is easy to learn and remember, efficient, visually pleasing, and fun to use and enables quick recovery from errors.	Effectiveness Efficiency Satisfaction Ease of use Visually pleasing Fun to use Few errors	Effectiveness Efficiency Satisfaction Satisfaction Memorability Satisfaction Security
Span et al [60], 2014	NR	User-friendliness User acceptance and satisfaction Participants’ appraisal of the DecideGuide for Making Decisions	Satisfaction Satisfaction Productivity
Støme et al [61], 2019	NR	Feasibility Ease of use Tasks on time Utility	Efficiency Satisfaction Productivity Universality
Van Maurik et al [65], 2019	Clinicians were asked to complete the SUS^g^ after using the tool.	Applicability User-friendliness Reliability	Effectiveness Satisfaction Memorability
Williams et al [67], 2016	NR	Learnability User control User empowerment Navigation Consistency Actionable feedback and available help	Memorability Productivity Productivity Memorability Memorability Memorability
Zafeiridi et al [68], 2020	Usability is measured as the user-friendliness (eg, ease to learn) and perceived usefulness in addressing users’ needs.	Usefulness Ease of use User satisfaction	Productivity Satisfaction Satisfaction

^a^As defined by the authors.

^b^HCV: hepatitis C virus.

^c^NR: not reported.

^d^ADAPT: Avoiding Diabetes Through Action Plan Targeting.

^e^ISO: International Organization for Standardization.

^f^NCI: National Cancer Institute.

^g^SUS: System Usability Scale.

### Technology for SDM

Table 2 presents the type of SDM technologies that were used across the included studies. Technologies for SDM included clinical decision support systems (9/33, 27%), mobile health apps (9/33, 27%), and web-based aids (15/33, 46%). The SDM context was mainly between clinicians and patients (32/36, 89%). The types of technology for SDM were mapped to usability parameters, including effectiveness (10/38, 26%), efficiency (11/38, 29%), memorability (20/38, 53%), satisfaction (27/38, 71%), security (5/38, 13%), universality (4/38, 10%), and productivity (16/38, 42%) based on Gupta et al [23]. The most common SDM technologies evaluated for usability were web-based aids. Satisfaction was the most common usability parameter mapped to types of SDM technologies.

**Table 2:** Technologies to support shared decision-making (SDM).

Study	Title of developed technology	Technology overview	Stage of development of technology intervention	Framework followed or guidelines by the authors	Description of SDM context or type of decision-making	Usability parameters measured	Gupta et al [23] framework
Anderson et al [32], 2014	STOP^a^ Tool	Web-based user interface for adaptive clinical decision support integrated into electronic health record	Preimplementation	Framework based on usability engineering	SDM between patients and clinicians for self-management and secondary stroke prevention	Ease of use Fun to use Navigation Understandability Visually pleasant User-friendly Efficient interaction	Satisfaction Satisfaction Memorability Memorability Memorability Satisfaction Efficiency
Barrio et al [33], 2017	SIDEAL^b^	Mobile app	Developmental laboratory	MI^c^ was the main source of guidance throughout the development process.	SDM between patients and clinicians related to self-management of alcohol dependence	Simplicity Ease of use User-friendly User control	Satisfaction Satisfaction Satisfaction Productivity
Bauerle Bass et al [34], 2018	“Take Charge, Get Cured”	mHealth^d^ decision support tool	Development	Model of illness self-regulation, information-communication theory, and formative evaluation framework	SDM between patients and clinicians related to initiating hepatitis C treatment	Visibility Ease of use Learnability Comprehensiveness	Memorability Satisfaction Memorability Memorability
Berry et al [35], 2015	P3P^e^	Web-based decision aid	Preimplementation	NR^f^	SDM between patients and clinicians about prostate cancer management options	Ease of use Readability	Satisfaction Memorability
Bogza et al [36], 2020	Web-based decision aids	—^g^	Development	User-centered approach; Center for eHealth and Wellbeing Research guidelines	SDM between patients and clinicians	Acceptability Satisfaction	Satisfaction
Burns and Pickens [37], 2017	NR	Technology-based CDSS^h^ for app-based assessments	Preimplementation	NR	SDM between providers, client, and family for home evaluation and modifications	Efficiency User control Consistency Feedback	Efficiency Productivity Memorability Productivity
Canally et al [38], 2015	NR	GUIs^i^	Developmental laboratory	NR	Shared decision support system that integrated biophysiological information obtained through multiple nonintrusive monitoring for home care	User-friendly Usefulness Feedback Navigation User control	Satisfaction Productivity Productivity Memorability Productivity
Chrimes et al [39], 2014	ADAPT^j^	Clinical decision support tool integrating evidence-based shared goal-setting components into electronic health record	Developmental laboratory	ADAPT framework	SDM between patients and clinicians for behavior changes to manage prediabetes	Ease of use Visually pleasing	Satisfaction Memorability
Cox et al [40], 2015	eCODES^k^	Web-based decision aid integrated into data entry and management system	Developmental laboratory	NR	SDM between clinicians and surrogate decision makers of patients receiving prolonged mechanical ventilation	Ease of use Simplicity	Satisfaction Satisfaction
Cuypers et al [41], 2019	Web-based decision aid system	—	Development	On the basis of existing evidence-based Canadian decision aid, developed by Feldman-Stewart et al [70-74]	SDM between patients and clinicians	NR	NR
Danial-Saad et al [43], 2016	OSCAR^l^	Interactive CDSS	Development laboratory	LUCID^m^ framework	Server-client system to recommend and select optimal pointing device	Visibility Minimizing errors Consistency Efficiency Memorability Affordance Feedback Effective use of language User control Flexibility Navigation Ease of use Naturalness User-friendly Ease of performance	Memorability Security Memorability Efficiency Memorability Universality Productivity Effectiveness Productivity Productivity Memorability Satisfaction Satisfaction Satisfaction Satisfaction Satisfaction
De Vito Dabbs et al [42], 2009	Pocket PATH^n^	IHT^o^ through handheld computer device	Preimplementation	User-centered design	SDM between patients of lung transplant and their transplant team about self-monitoring of critical values	User control Action feedback Ease of use	Productivity Productivity Satisfaction
Fleisher et al [44], 2008	CONNECT^p^	Interactive web-based communication aid	Preimplementation	C-SHIP^q^ model	SDM between patients and clinicians about treatment decisions supported through communication skill development modules	Readability Simplicity Visually pleasing	Memorability Satisfaction Memorability
Flynn et al [45], 2015	COMPASS^r^ prototype	User interface with decision analytical model developed on iPad mobile device	Developmental laboratory	Decision analytic model predictions developed from S-TPI^s^	SDM between clinicians and patients about patient-specific treatment options for acute ischemic stroke and personalized information to patients	User-friendly Effective use of language Visibility Efficient interaction	Satisfaction Effectiveness Memorability Efficiency
Fu et al [46], 2020	Mobile apps	—	Testing	Nielsen heuristics	Unclear	Satisfaction Effectiveness Efficiency	Satisfaction Effectiveness Efficiency
Goud et al [47], 2008	CARDSS^t^	Guideline-based computerized decision support systems	Implementation	Clinical guidelines	SDM between clinicians and patients for patient-specific care for cardiac rehabilitation and patient management	Effectiveness Minimizing errors	Effectiveness Security
Grim et al [48], 2017	NR	Interactive web-based software	Preimplementation	The team followed published evidence on the consensus guidelines for development of decision aids and SDM.	SDM between patients and clinicians about care in psychiatric services	Ease of use User-friendly	Satisfaction Satisfaction
Holch et al [49], 2017	e-RAPID^u^	Integrated electronic platform for patient self-report	Preimplementation	Translational research model	SDM between patients and clinicians for management of events during cancer treatment	User interface Action feedback Visually pleasing	Productivity Productivity Memorability
Jameie et al [50], 2019	Cardiac telerehabilitation platform	—	Development	BACPR^v^	SDM between patients and clinicians	NR	NR
Jessop et al [51], 2020	“Take Charge, Get Cured”	mHealth treatment decision support tool embedded in Articulate 360 app	Preimplementation	NR	SDM between patients and physicians about hepatitis C treatment	NR	—
Kallen et al [52], 2012	PRO^w^-based Palliative and Hospice Care Management System—prototype	Electronic PRO system	Implementation	User-centered design approach	SDM between patients in palliative care and treating physician or nurse	Efficiency Interface quality Navigation Simplicity Visually pleasing	Efficiency Efficiency Memorability Satisfaction Memorability
Li et al [53], 2013	ANSWER^x^	Web-based decision aid with educational modules	Preimplementation	The International Patient Decision Aid Standards and the Jabaja-Weiss edutainment decision aid model	SDM between patients and clinicians about using methotrexate	Visually pleasing Efficient interaction	Memorability Efficiency
Murphy et al [54], 2020	CP-PDA^y^	Web-based algorithmic intervention	Development	International Patient Decision Aid Standards criteria checklist, SUNDAE^z^ checklist, and the EQUATOR^a^^a^ CONSORT^a^^b^ checklist	SDM between patients and clinicians about postprostatectomy care regarding continence product choice	Visibility Clarity Ease of use Usefulness Comprehensibility Acceptability	Memorability Memorability Satisfaction Productivity Memorability Satisfaction
Rochette et al [55], 2008	StrokEngine-Family	Stroke rehabilitation layperson website	Implementation	NR	SDM between patients and clinicians	Ease of use Simplicity	Satisfaction Satisfaction
Setiawan et al [57], 2019	iMHere^ac^ 2.0	Adaptive mHealth system with mobile app modules (client app, caregiver app, web-based clinician portal, back-end server, and 2-way communication protocol)	Development	User-centered design	Monitoring and support of self-management for people with chronic conditions and disabilities and allowing for personalized and adaptive treatment strategies	Error prevention User satisfaction Ease of use Usefulness	Security Satisfaction Satisfaction Productivity
Schön et al [56], 2018	Digital interactive decision support tool	—	Development	The decision support tool is based on the theoretical framework of SDM.	SDM between patients and clinicians	Ease of use User-friendly	Satisfaction Satisfaction
Snyder et al [58], 2009	PatientViewpoint prototype	Web-based system to collect PROs linked with electronic medical record	Preimplementation	NR	SDM between patients and clinicians for cancer management	Ease of use Efficient interaction	Satisfaction Efficiency
Span et al [60], 2014	DecideGuide	Interactive web tool	Developmental laboratory	NR	SDM in dementia care networks between patients, care managers, and informal caregivers	Simplicity Ease of use Functionality Visibility User control Readability Social acceptability	Satisfaction Satisfaction Universality Memorability Productivity Memorability Universality
Span et al [59], 2018	DecideGuide	Interactive web tool	Preimplementation	The 5 phases of the CeHRes^ad^ road map	SDM made by care network of people with dementia (patients, care managers, and informal caregivers)	Social acceptability Minimize error Efficiency Action feedback	Universality Security Efficiency Productivity
Støme et al [61], 2019	Vett interactive mobile app	—	Implementation	NR	Unclear	Feasibility Ease of use Tasks on time Utility	Efficiency Satisfaction Productivity Universality
Tony et al [62], 2011	EVIDEM^a^^e^ decision support framework	MCDA^a^^f^ and HTA^a^^g^	Developmental laboratory	EVIDEM framework	SDM between patients and clinicians to appraise health care interventions	Learnability	Memorability
Toth-Pal et al [63], 2008	EviBase	CDSS through internet-based application	Implementation	Clinical guidelines (1 Swedish and 2 European)	SDM between clinicians and patients through integration of individual patient data with guidelines for management of chronic heart failure	Flexibility Ease of use	Effectiveness Satisfaction
Tsai et al [64], 2019	MagicPlan	Mobile app with laser distance measurer	Preimplementation	NR	Clinical home evaluations with virtual floor plan for DME^ah^ recommendations	Visibility Ease of use Error prevention Usefulness Satisfaction	Memorability Satisfaction Security Productivity Satisfaction
Van Maurik et al [65], 2019	Web-based diagnostic support tool named ADappt	—	Development	NR	SDM between patients and clinicians	Applicability User-friendliness Reliability	Effectiveness Satisfaction Memorability
Welch et al [66], 2015	MedMinder	Cellular pillbox monitoring device	Implementation	NR	SDM between clinicians and patients related to treatment and adherence support	Efficient interaction Action feedback Readability	Efficiency Productivity Memorability
Williams et al [67], 2016	NR	Clinical decision support on mHealth app	Developmental laboratory	User-centered design approach (user interface and user experience design)	SDM between clinicians and patients for patient-specific recommendations for cardiovascular disease	Actionable feedback Interface quality Information quality User empowerment Simplicity Ease of use Readability Efficiency Practicality	Productivity Efficiency Memorability Productivity Satisfaction Satisfaction Memorability Efficiency Effectiveness
Zafeiridi et al [68], 2018	CAREGIVERSPRO-MMD^ai^	Web-based platform	Development	User-centered design	Social network for sharing information, tips, and support across peers and health professionals	Usefulness Ease of use User satisfaction	Productivity Satisfaction Satisfaction
Zheng et al [69], 2017	NR	mHealth app with PROs	Preimplementation	User-centered design principles	SDM between patients and clinicians for knee arthritis treatment	Action feedback Interface quality Interface information Visually pleasing	Productivity Efficiency Effectiveness Memorability

^a^STOP: Self-Management to Prevent Stroke.

^b^SIDEAL: Soporte Innovador al paciente con Dependencia del Alcohol, Innovative Support to the Alcohol Dependent Patient.

^c^MI: motivational interviewing.

^d^mHealth: mobile health.

^e^P3P: The Personal Patient Profile-Prostate.

^f^NR: not reported.

^g^Data not available.

^h^CDSS: clinical decision support system.

^i^GUI: graphical user interface.

^j^ADAPT: Avoiding Diabetes Through Action Plan Targeting.

^k^eCODES: Electronic Collaborative Decision Support.

^l^OSCAR: Ontology-Supported Computerized Assistive Technology Recommender.

^m^LUCID: logical user-centered interaction design.

^n^PATH: Personal Assistant for Tracking Health.

^o^IHT: interactive health technology.

^p^CONNECT: web-based communication aid.

^q^C-SHIP: Cognitive-Social Health Information Processing.

^r^COMPASS: Computerized Decision Aid for Stroke Thrombolysis.

^s^S-TPI: Stroke-Thrombolytic Predictive Instrument.

^t^CARDSS: Cardiac Rehabilitation Decision Support System.

^u^e-RAPID: Electronic Patient Self-Reporting of Adverse-Events: Patient Information and Advice.

^v^BACPR: British Association for Cardiovascular Prevention and Rehabilitation.

^w^PRO: patient-reported outcome.

^x^ANSWER: Animated, Self-Serve, Web-Based Research Tool.

^y^CP-PDA: Continence Product Patient Decision Aid.

^z^SUNDAE: Standards for Universal Reporting of Patient Decision Aid Evaluations.

^aa^EQUATOR: Enhancing the Quality and Transparency of Health Research.

^ab^CONSORT: Consolidated Standards of Reporting Trials.

^ac^iMHere: Interactive Mobile Health and Rehabilitation.

^ad^CeHRes: Center for eHealth Research and Disease Management.

^ae^EVIDEM: Evidence and Value: Impact on Decision-Making.

^af^MCDA: multicriteria decision analysis.

^ag^HTA: health technology assessment.

^ah^DME: durable medical equipment.

^ai^CAREGIVERSPRO-MMD: Caregivers Patient-Reported Outcome-Mild Mental Disorder.

### Usability Evaluation Methods

**Figure 2:**
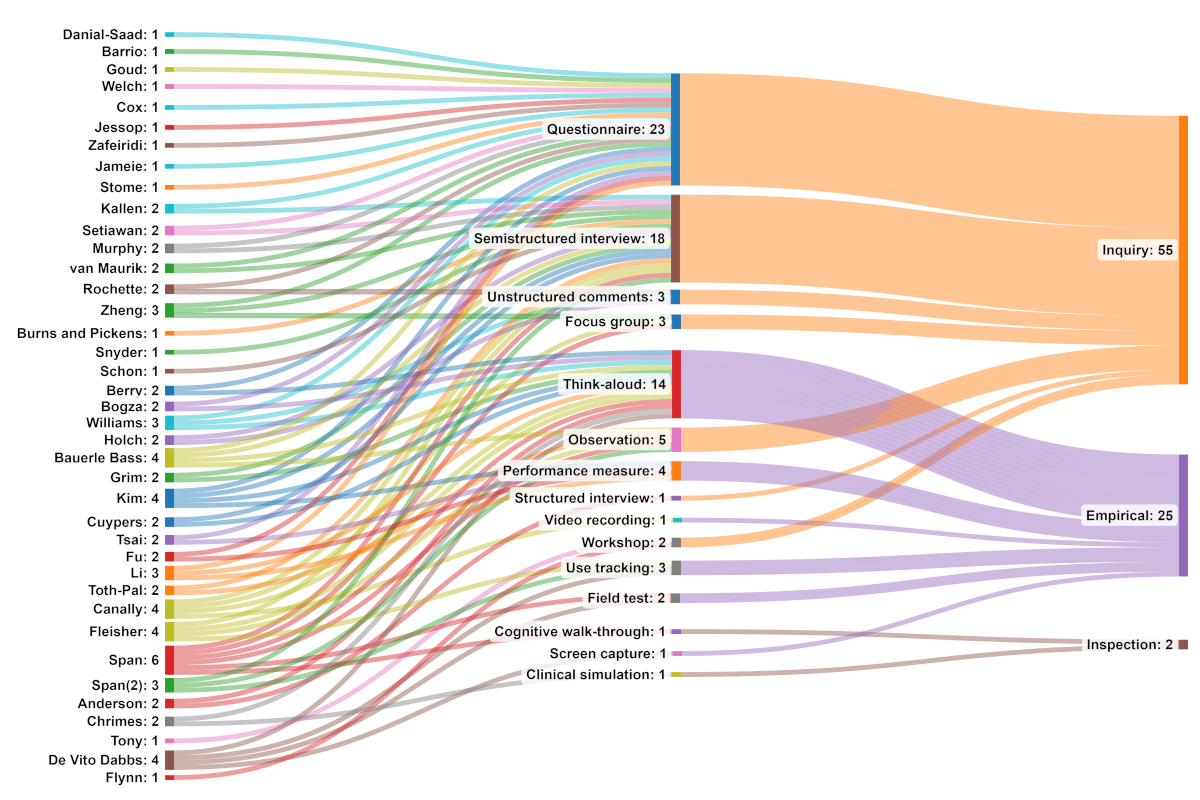
Usability evaluation methods [32-69].

**Figure 3:**
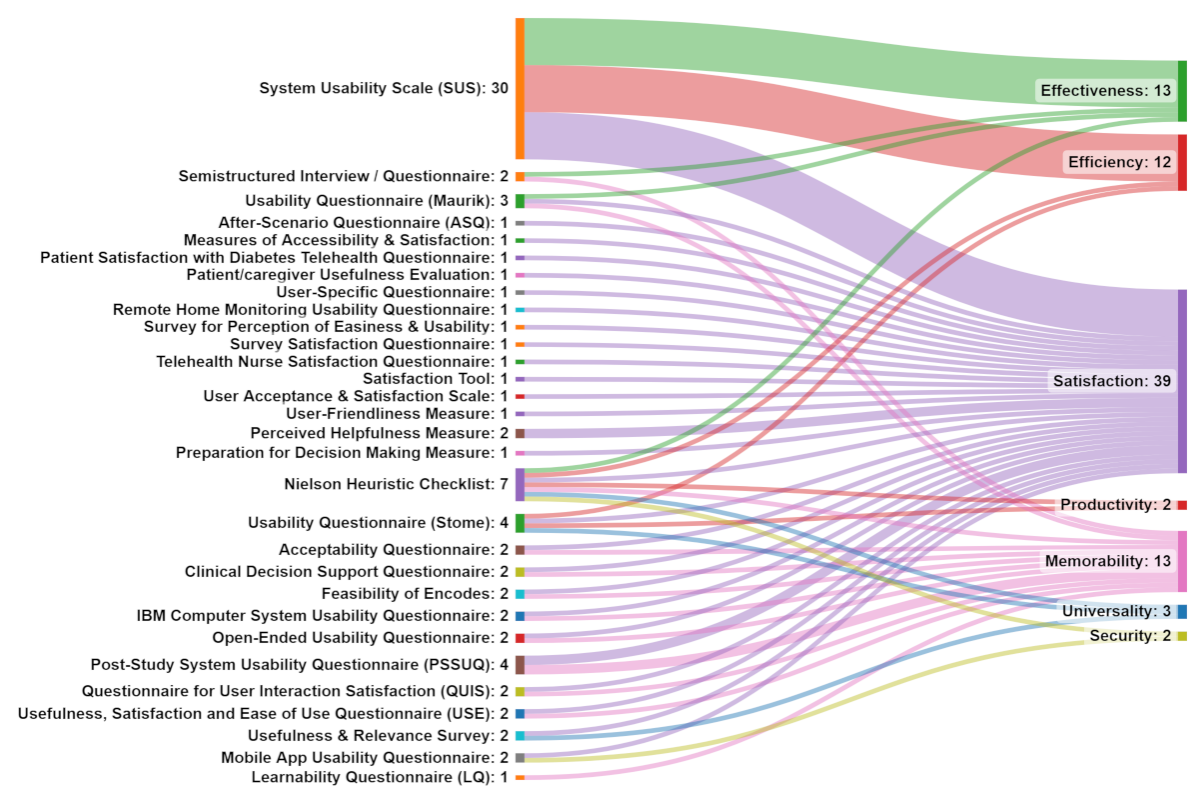
Mapping the usability evaluation methods to usability parameters based on a comprehensive hierarchal usability model presented by Gupta et al [23].

The usability evaluation methods were categorized, based on the framework by Jacobsen [31], into (1) empirical (think-aloud protocol, 14/38, 36%; user tracking, 3/38, 8%; performance measures, 4/38, 10%; field test, 2/38, 5%; video recording, 1/38, 2%; and screen capture, 2/38, 5%), (2) inspection (cognitive walk-through, 1/38, 2% and *Near live* clinical situation, 1/38, 2%), and (3) inquiry (focus groups, 3/38, 8%; workshops, 2/38, 5%; semistructured interviews, 16/38, 42%; structured interviews, 1/38, 2%; questionnaires, 24/38, 63%; observations, 5/38, 13%; and comments, 3/38, 8%; Table 3). An important point to emphasize is the frequency with which researchers used 1 (13/38, 34%), 2 (15/38, 39%), 3 (7/38, 18%), 4 (2/38, 5%), and 6 (1/38, 2%) methods from the framework by Jacobsen [31], presented in Figure 2 [32-69]. Most (28/38, 73%) used 1 or 2 methods of evaluation. Usability was assessed during development (18/38, 47%), preimplementation (13/38, 34%), or implementation (7/38, 18%) through a variety of measures, including usability questionnaires (15/38, 39%), tailored tools developed by the authors (17/38, 45%), and acceptance and satisfaction questionnaires (6/38, 16%). The usability evaluation parameters identified by the authors were mapped to the usability parameters explained by Gupta et al [23], including effectiveness (13/38, 34%), efficiency (12/38, 31%), memorability (13/38, 34%), productivity (2/38, 5%), security (2/38, 5%), and satisfaction (32/38, 84% Figure 3 and Table 4).

**Table 3:** Usability evaluation methods.

Study	Method	Jacobsen [31] framework	Details
Anderson et al [32], 2014	Think-aloud protocol Structured interview	Empirical Inquiry	Think-aloud method using prototype and scripted test case scenario Structured interview with open-ended questions (feedback on barriers and facilitators and usefulness)
Barrio et al [33], 2017	Questionnaire	Inquiry	USE^a^ questionnaire
Bauerle Bass et al [34], 2018	Think-aloud protocol Semistructured interview Questionnaire	Empirical Inquiry Inquiry	Think-aloud method when following navigational steps (audiotaped with observation notes) Semistructured interviews (feedback on graphics, voiceover, content, and purpose) Usefulness and relevance survey
Berry et al [35], 2015	Think-aloud protocol Questionnaire	Empirical Inquiry	Think-aloud session while interacting with website, with probing questions Acceptability questionnaire
Bogza et al [36], 2020	Think-aloud protocol Questionnaire	Empirical Inquiry	Think-aloud method while reviewing web-based decision aid (probing questions) Ottawa Acceptability Questionnaire and SUS^b^ questionnaire
Burns and Pickens [37], 2017	Semistructured interviews	Inquiry	Semistructured interview on perceptions of process and technology needs
Canally et al [38], 2015	Semistructured interviews Think-aloud methodology Video recording of computer screen Focus groups	Inquiry Empirical Empirical Inquiry	Open-ended questions about functions and areas of improvement Think-aloud method with prototype using simulated case developed with clinician Video recording of screen interactions Series of focus groups to refine the instrument
Chrimes et al [39], 2014	Think-aloud protocol through scripted scenario “Near live” clinical stimulation Screen capture recording	Empirical Inspection Empirical	Think-aloud session with scripted navigation instructions for prediabetes counseling scenario Clinical stimulation without navigational guidance mimicking clinical workflows Motion screen capture for onscreen recordings
Cox et al [40], 2015	Questionnaire	Inquiry	SUS and ASQ^c^
Cuypers et al [41], 2019	Think-aloud protocol Semistructured interviews	Empirical Inquiry	Think-aloud method while navigating the decision aid Semistructured interview following 30 minutes of navigating the decision aid
De Vito Dabbs et al [42], 2009	Think-aloud protocol Field test Screen capture technology Use tracking	Empirical Empirical Empirical Empirical	Think-aloud session with paper prototype and scenarios (iterative testing of features) Field test to assess the percentage of features that users accessed Data capture and use tracking of tool features (hits per feature, percentage of measurements recorded and transmitted, and times users contacted clinicians when prompted by message) ASQ and PSSUQ^d^
Danial-Saad et al [43], 2016	Questionnaire	Inquiry	SUS questionnaire
Fleisher et al [44], 2008	Think-aloud protocol Interviews Use tracking	Empirical Inquiry Empirical	Think-aloud session while reviewing the site, with observations Interview questions Use tracking of program (use of “help” button and number of warning messages)
Flynn et al [45], 2015	Interactive group workshops	Inquiry	Interactive group workshops with stroke clinicians and patients or relatives with paper prototype and functional prototype (feedback on appearance, layout, and features)
Fu et al [46], 2020	Performance measure Questionnaire	Empirical Inquiry	Checklist for intuitive design modified for diabetes apps, originally adapted from the 10 heuristics by Nielsen used for a healthy eating app evaluation SUS questionnaire
Goud et al [47], 2008	Questionnaire	Inquiry	CSUQ^e^ questionnaire
Grim et al [48], 2017	Think-aloud protocol Semistructured interviews	Empirical Inquiry	Think-aloud method with paper prototype, with observation of behavior (video recording and field notes) Semistructured interview following protocol guide
Holch et al [49], 2017	Semistructured interviews Written comments	Inquiry Inquiry	Semistructured interviews about the experience Written comments about logging in, navigating the system, and accessing features
Jameie et al [50], 2019	Questionnaire	Inquiry	SUS questionnaire
Jessop et al [51], 2020	Questionnaire	Inquiry	PrepDM^f^ scale with added items on perceived usefulness and user-friendliness
Kallen et al [52], 2012	Interviews Questionnaire	Inquiry Inquiry	Interviews with physicians or nurses and patients or caregivers to understand their needs and requirements as to the use of a computer system to help them manage their daily clinical activities, especially regarding the use of PRO^g^ assessments in patient care Providers used the prototype system to complete 2 assessments, the Memorial Delirium Assessment Scale and the Edmonton Classification System for Cancer Pain; patients and caregivers used the electronic PRO system to complete the Edmonton Symptom Assessment System and the Cut down, Annoyed, Guilty, and Eye-opener assessments
Li et al [53], 2013	Think-aloud protocol Questionnaire Performance measure	Empirical Inquiry Empirical	Think-aloud method when navigating decision aid, with probing questions (audio recorded and field notes) SUS questionnaire Time to complete the tool (minutes)
Murphy et al [54], 2020	Questionnaire Semistructured interview	Inquiry Inquiry	Questionnaire developed for the study for feedback on prototype for alpha testing Semistructured interviews with clinicians about usefulness and usability of final prototype in clinical practice
Rochette et al [55], 2008	Questionnaire Open-ended questions	Inquiry Inquiry	Questionnaire developed for the study combining open-ended questions whenever a score of dissatisfaction was given on a closed-ended question
Schön et al [56], 2018	Semistructured interview	Inquiry	Semistructured interview guide followed in focus groups (feedback on use of tool, usability, and impact on care planning and decision-making)
Setiawan et al [57], 2019	Questionnaire Semistructured interview	Inquiry Inquiry	PSSUQ following requested tasks on app Semistructured interview for further comments and suggestions
Snyder et al [58], 2009	Semistructured interviews	Inquiry	Semistructured interview while presenting a mock-up of the web application (feedback on features)
Span et al [60], 2014	Focus group sessions Cognitive walk-through Think-aloud method Field test Semistructured interviews Observation	Inquiry Inspection Empirical Empirical Inquiry Inquiry	Sketches using paper-based mock prototype presented to focus group Cognitive walk-through of interactive prototype to identify possible user problems Think-aloud method while using tool on tablet at home Field test of final prototype to assess user-friendliness, satisfaction, and value placed on tool Structured interviews throughout field-testing In-person observation of use of tool during field-testing
Span et al [59], 2018	Semistructured interviews Observations Use tracking	Inquiry Inquiry Empirical	Semistructured interviews (feedback on satisfaction, usefulness, user-friendliness, and use for decision-making) Observations of use during case manager home visits with people with dementia Use tracking of logged information (frequency of use and topics)
Støme et al [61], 2019	Questionnaire	Inquiry	Usability questionnaire developed for the study administered to patients
Tony et al [62], 2011	Workshop sessions	Inquiry	NR^h^
Toth-Pal et al [63], 2008	Semistructured interviews Observation	Inquiry Inquiry	Semistructured interviews after training and field test Field observations of patient visits following predefined guide (use and communication)
Tsai et al [64], 2019	Questionnaire Performance measure	Inquiry Empirical	Questionnaires developed for the study for lay participants and clinicians Time needed to finish a floor plan using the mobile app
Van Maurik et al [65], 2019	Interviews Questionnaire	Inquiry Inquiry	Interviews about prototype with patients and caregivers with software developer (feedback on storyline and graphics) Usability questionnaire developed for the study (administered to providers) and SUS questionnaire
Welch et al [66], 2015	Questionnaires	Inquiry	Patient questionnaire on remote home monitoring device usability, patient satisfaction with the diabetes telehealth program, primary care provider feedback on the clinical decision support report, and telehealth nurse satisfaction with the program Questionnaires developed for the study for patients (feedback on device usability and satisfaction), primary care providers (feedback on clinical decision support), and telehealth nurse (feedback on satisfaction)
Williams et al [67], 2016	Think-aloud protocol Unstructured comments Questionnaire	Empirical Inquiry Inquiry Inquiry	Think-aloud method one-on-one for test cases (audio recording of verbal feedback) Immediate unstructured comments provided via email, telephone, or SMS text message Open-ended questions about use (amount, type of visits, and components used) SUS questionnaire
Zafeiridi et al [68], 2018	Questionnaire Open-ended comments	Inquiry Inquiry	Questionnaire developed for the study Open-ended questions about the improvement of the platform were asked when participants provided low scores for a feature or function
Zheng et al [69], 2017	Focus groups Interviews Questionnaire	Inquiry Inquiry Inquiry	Patient focus groups (feedback on experience, assessment of interface, preferences on presentation, and use for treatment decision-making) Clinician interviews (feedback on expectations from individualized PRO report) Survey developed for the study on perception of easiness and usability of interfaces

^a^USE: Usefulness, Satisfaction and Ease of Use.

^b^SUS: System Usability Scale.

^c^ASQ: After-Scenario Questionnaire.

^d^PSSUQ: Post-Study System Usability Questionnaire.

^e^CSUQ: Computer System Usability Questionnaire.

^f^PrepDM: Preparation for Decision Making.

^g^PRO: patient-reported outcome.

^h^NR: not reported.

**Table 4:** Usability measures.

Usability measures	Type of scale	Items	Usability evaluation parameters identified by the authors	Gupta et al [23] framework
Acceptability questionnaire [56]	5-point Likert scale	How easy was the program for you to use? How understandable were the questions? How much did you enjoy using the program? How helpful was it to complete the program? Was the amount of time it took to complete the program acceptable? How valuable was the information? Overall, how would you rate your satisfaction with this program? Please rate the usefulness to you of: “your part in the decision” section. Please rate the usefulness to you of: “information topics” section. Please rate the usefulness to you of: “information on statistics” section. Please rate the usefulness to you of: video clips. Please rate the usefulness to you of: prostate cancer internet sites.	Ease of use Learnability	Satisfaction Memorability
ASQ^a^ [45]	7-point Likert scale	Ease of completing tasks in scenario Time to complete tasks Support when completing tasks	Ease of use	Satisfaction
Clinical decision support report questionnaire [53]	5-point Likert scale	Clear and easy to understand: Medication adherence percentages Medication adherence calendars BG^b^ graphs BP^c^ graphs Detailed logs of BP and BG readings Clinically useful: Medication adherence percentages Medication adherence calendars BG graphs BP graphs Detailed logs of BP and BG readings For my patients, I want this report in the EMR^d^ For my patients, I want this report in hard copy	Ease of use Visibility	Satisfaction Memorability
Feasibility of encodes [55]	—^e^	What did you like most about the encodes program? Easy information, particularly if you have no experience in this situation Easy to use It puts it in black and white It focuses the question at hand on the patient User-friendly I liked how patient- and family-centered it was Informative Interactive iPad is a familiar platform Wording is simple What did you dislike most about the encodes program? Touch screen Sensitive topic Does not make decisions for you Simplistic Delay How could the encodes program be improved? Would like even more information about prognosis Make [the information] more complex Make forward button more obvious Even more illustrations Make more options focusing on each specific patient’s case	Ease of use Visibility User-friendly	Satisfaction Memorability Satisfaction
IBM CSUQ^f^ [39]	7-point Likert scale	Satisfaction and ease of use Overall, I am satisfied with how easy it is to use this system It is simple to use this system I can effectively complete my work using this system I am able to complete my work quickly using this system I am able to efficiently complete my work using this system I feel comfortable using this system It was easy to learn to use this system I believe I became productive quickly using this system Quality and clarity of information The system gives error messages that clearly tell me how to fix problems It is easy to find the information I need The information provided with the system is easy to understand The information is effective in helping me complete my work The organization of the information on the system screens is clear System’s interface The interface of this system is pleasant I like using the interface of this system This system has all the functions and capabilities I expect it to have	Satisfaction Visibility Ease of use	Satisfaction Memorability Satisfaction
LQ^g^ [32]	6-point Likert scale	The system reminded me of the important information needed for the pointing device adaptation process for people with disabilities The organization of the information helped me arrange the stages of prescribing a pointing device for people with disabilities The organization and the display of the information helped my clinical reasoning The system provided me with new information for the pointing device adaptation process for people with disabilities The system offered me information that made me change my pointing device adaptation plan The system concentrated the professional language and terminology used in the pointing device adaptation process	Learnability	Memorability
Measures of accessibility and satisfaction [55]	5-point Likert scale	Acceptability I was able to answer the questions in the program I was able to complete the computer program Satisfaction I was satisfied with the computer program overall I was satisfied with how easy it was to use the program I was satisfied with the layout of the program I was satisfied with the instructions Feasibility Prefer printed version of decision aid	Satisfaction User-friendly	Satisfaction Satisfaction
Mobile app usability concept questions [64]	5-point Likert scale	Please rate the user interface Please rate the ease of use Please rate the clarity of error messages Please rate how useful the mobile app is overall How likely would you recommend using this mobile app for home evaluation?	Visibility Ease of use Error prevention Usefulness Satisfaction	Memorability Satisfaction Security Productivity Satisfaction
Nielsen heuristic checklist [46]	Likert scale	The heuristic checklist has 10 intuitive design principles, and the severity of each violation is rated as minor, moderate, major, or catastrophic (1-4).	Help and documentation Error prevention Esthetic and minimalist design Flexibility and efficiency of use Recognition rather than recall Match between app and the real world User control and freedom Consistency and standards Feedback and visibility Helps recover from errors	Productivity Security Satisfaction Efficiency Memorability Efficiency Universality Universality Effectiveness Productivity
Open-ended questionnaire for usability [32]	—	Would you use the system to support your clinical decision reasoning process? Describe 1 or 2 new things you have learned following the use of the system Suggest 1 or 2 features you would add to the system Please add your comments and suggestions	Learnability Ease of use	Memorability Satisfaction
Patient satisfaction with diabetes telehealth program questionnaire [53]	5-point Likert scale	Happy with RHM^h^ device training before program Felt supported by diabetes care team Nurse phone calls were helpful Nurse calls lasted a good amount of time Liked getting help at home over the phone Would recommend program to other patients with T2D^i^ Would keep using this program at home	Satisfaction	Satisfaction
Perceived general helpfulness and value [51]	3-point Likert scale	How helpful was the material? Would you recommend it to others? How clear was the information?	Usefulness	Productivity
Perceived helpfulness [51]	10-point Likert scale	The information about hepatitis C was helpful The video of other people talking about their experiences was helpful The part where I was able to choose questions to talk with my doctor about was helpful The voiceover information with pictures about HCV^j^ was helpful The part where I mark how likely I was to be treated was helpful The summary at the end was helpful	User-friendliness Usefulness	Satisfaction Productivity
Perceived usefulness [51]	10-point Likert scale	App provided new information App helped me feel prepared to talk with doctor App helped with my emotional concerns App increased my knowledge App help me be less anxious	Usefulness	Productivity
PSSUQ^k^ [45,57]	7-point Likert scale	Easy-to-use system Simple-to-use system Effectively complete tasks and scenarios Quickly complete tasks and scenarios Efficiently complete tasks and scenarios Comfort using system Easy to learn to use system Belief one could become productive using the system Error messages were clear Easily recover from mistakes Information about system was clear Easy to find needed information Easy-to-understand information Information helped complete the task Information was clearly organized Interface was pleasant Enjoyed using interface	User satisfaction Ease of use Learnability Visibility User-friendly	Satisfaction Satisfaction Memorability Memorability Satisfaction
PrepDM^l^ scale [51]	5-point Likert scale	App helped—recognize that a decision about HCV treatment needs to be made App prepared—to make a better decision about HCV treatment App helped—think about the pros and cons of HCV treatment App helped—know that decision about treatment depends on what matters most to me App helped—organize your own thoughts about the HCV treatment decision App helped—identify questions you want to ask your doctor App prepared—to talk to your doctor about what matters most to you App prepared—for a follow-up visit with your doctor	Usefulness	Productivity
Remote home monitoring device usability questionnaire [53]	5-point Likert scale	Pillbox: Easy to use Helped organize medications Using it easily fit into daily routines Ability to set it up in a convenient place at home Easy to understand how to refill BG meter: Easy to use Display was clear and easy to read Using it easily fit into daily routines Ability to set it up in a convenient place BP meter: Easy to use Encouraged me to take BP more often Using it easily fit into daily routines Ability to set it up in a convenient place	Ease of use	Satisfaction
Survey for perception of easiness and usability of the 6 interfaces [43]	5-point Likert scale	Vertical response options (user interface 1) Horizontal response options (user interface 2) Vertical response options with a movable slide (user interface 3) Horizontal response options with a movable slide (user interface 4) 3-point multimapping (user interface 5) 6-point multimapping (user interface 6)	Ease of use	Satisfaction
Survey satisfaction questionnaire [46]	Likert scale	Preconsultation survey How much time on the internet per week? <1 hour, 1-4 hours, 5-7 hours, 8-14 hours, and >14 hours Length of survey: reasonable, a little long, and much too long Satisfaction with survey: not very satisfied, slightly satisfied, moderately satisfied, and extremely satisfied Where they completed the survey: home, work, friend and family, public computer, resource education center on-site, and other Postconsultation survey: not at all, a little, moderately, quite a lot, and extremely How helpful was the survey to the consultation How helpful was the module to the consultation Did you feel the survey affected how you communicated with your physician? Did you feel the skills module survey affected how you communicated with your physician? Which was more helpful? Did you feel that taking part in the program helped your communication with your doctor?	Satisfaction	Satisfaction
Telehealth nurse satisfaction questionnaire [53]	5-point Likert scale	Device training Web-based dashboard training Ability to contact patients by phone Ability to track DSM^m^ of patients Ability to work as a team with PCPs^n^ Overall satisfaction with telehealth program	Satisfaction	Satisfaction
Tool developed by the authors focused on description [52]	5-point Likert scale	Home page Easy to find Satisfaction with visual presentation (organization or content) Satisfaction with appearance of text (size, type of writing, and spacing) and satisfaction with colors Module 1 Easy to find Satisfaction with visual presentation (organization or content) Satisfaction with appearance of text (size, type of writing, and spacing) Usefulness of information Module 2 Easy to find Usefulness of information General appreciation Satisfaction with general appearance Easy to use Satisfaction with time required to open pages How user-friendly Overall satisfaction	Ease of use User-friendly Satisfaction	Satisfaction Satisfaction Satisfaction
Usability questionnaire [61]	100-point Likert scale	Vett on mobile phone is simple and intuitive to use Reminders of tasks arrive at the agreed-upon time It is easy and intuitive to answer the reminders It is easy and intuitive to answer that the task is done	Feasibility Ease of use Tasks on time Utility	Efficiency Satisfaction Productivity Universality
Usability questionnaire [65]	10-point Likert scale	Is it clear where ADappt could be used for (scale from 1-10)? How user-friendly would you rate this tool to be (scale from 1-10)? How reliable would you rate ADappt to be (scale from 1-10)? Would you use the final version of ADappt in your daily clinical routine (percentage of “yes”)?	Applicability User-friendliness Reliability	Effectiveness Satisfaction Memorability
Usability scale (SUS) questionnaire [32,36,40,46-48,50,55,57,65]	5-point Likert scale	I think that I would like to use this CDS^o^ app frequently. I found the CDS app unnecessarily complex. I thought the CDS app was easy to use. I think that I would need the support of a technical person to be able to use this CDS app. I found that the various functions in this CDS app were well integrated. I thought there was too much inconsistency in this CDS app. I would imagine that most people would learn to use this CDS app very quickly. I found the CDS app very cumbersome to use. I felt very confident using the CDS app. I needed to learn a lot of things before I could get going with this app.	Effectiveness Efficiency Satisfaction	Effectiveness Efficiency Satisfaction
USE^p^ questionnaire [37]	7-point Likert scale	Usefulness It helps me be more effective It helps me be more productive It is useful It gives me more control over the activities in my life It makes the things I want to accomplish easier to get done It saves me time when I use it It meets my needs It does everything I would expect it to do Ease of use It is easy to use It is simple to use It is user-friendly It requires the fewest steps possible to accomplish what I want to do with it It is flexible Using it is effortless I can use it without written instructions I do not notice any inconsistencies as I use it Both occasional and regular users would like it I can recover from mistakes quickly and easily I can use it successfully every time Ease of learning I learned to use it quickly I easily remember how to use it It is easy to learn to use it I quickly became skillful with it Satisfaction I am satisfied with it I would recommend it to a friend It is fun to use It works the way I want it to work It is wonderful I feel I need to have it It is pleasant to use	Usefulness Ease of use Ease of learning Satisfaction	Satisfaction Satisfaction Memorability Satisfaction
Usefulness and relevance survey [34]	7-point Likert scale	Satisfied with ease Simple to use Understand how to go from one screen to another Easy to choose which parts I want I felt comfortable using it Information was clear and easy Easy to find information I need Information effective for decision-making Tablet was easy to use Length of tool was right Right amount of information on hepatitis C Tool slanted toward convincing me Tool helpful for patients seeking information Tool helped me talk with doctor Videos and visuals were helpful	Usefulness Relevance	Productivity Productivity
User acceptance and satisfaction scale [49]	5-point Likert scale	All participants valued the tool positively; concerns about guide being too confronting Participants’ appraisal of the tool for making decisions—supportive tool Short lines of communication, awareness of the steps in decision-making, and improvements for the tool	User acceptance Satisfaction	Satisfaction Satisfaction
User-friendliness measured with an instrument based on the CeHRes^q^ assessment of design quality [49]	—	Ease of use: chat function easy for all Deciding together function too difficult for all Technical failures: problems with IT and internet connection Nice to have notifications, agenda, photos, memory games, and ability to send message to 1 person	User-friendliness Ease of use	Satisfaction Satisfaction
User-specific evaluation questionnaire for clinicians [47]	—	This system could improve our operational efficiency This system could help us improve our quality of patient care This system could help us better use patient assessments in clinical decision-making and patient care This system could help us identify important causal and temporal relationships between care events and outcomes that can aid our clinical decision-making This system could help us monitor patient status and better serve their needs This system will work well with our existing workflow This system will improve patient-provider communication This system could facilitate communication among members of a multidisciplinary team I will recommend our practice to adopt this system when it is fully developed I will recommend other practices to adopt this system when it is fully developed	Usefulness	Satisfaction
User-specific evaluation questionnaire for patients and caregivers [47]	—	I enjoyed using this system to report symptom status It is easy to complete patient assessments using this system This system can help me better use patient symptom status reports to communicate with health care providers This system can help me better use patient symptom status reports in decision-making about patient care This system can help in the monitoring of patient status to better serve patient needs It will be easier to use this system to complete patient assessments than to complete assessments using paper and pencil I would like to be a beta tester of this system when it is ready I would likely recommend that patient care providers adopt this system when it is fully developed	Usefulness	Satisfaction

^a^ASQ: After-Scenario Questionnaire.

^b^BG: blood glucose.

^c^BP: blood pressure.

^d^EMR: electronic medical record.

^e^Data not available.

^f^CSUQ: Computer System Usability Questionnaire.

^g^LQ: learnability questionnaire.

^h^RHM: remote home monitoring.

^i^T2D: type 2 diabetes.

^j^HCV: hepatitis C virus.

^k^PSSUQ: Post-Study System Usability Questionnaire.

^l^PrepDM: Preparation for Decision-Making.

^m^DSM: diabetes self-management.

^n^PCP: primary care provider.

^o^CDS: clinical decision support.

^p^USE: Usefulness, Satisfaction, and Ease of Use.

^q^CeHRes: Center for eHealth Research and Disease Management.

### Frameworks and Theoretical Models

**Figure 4:**
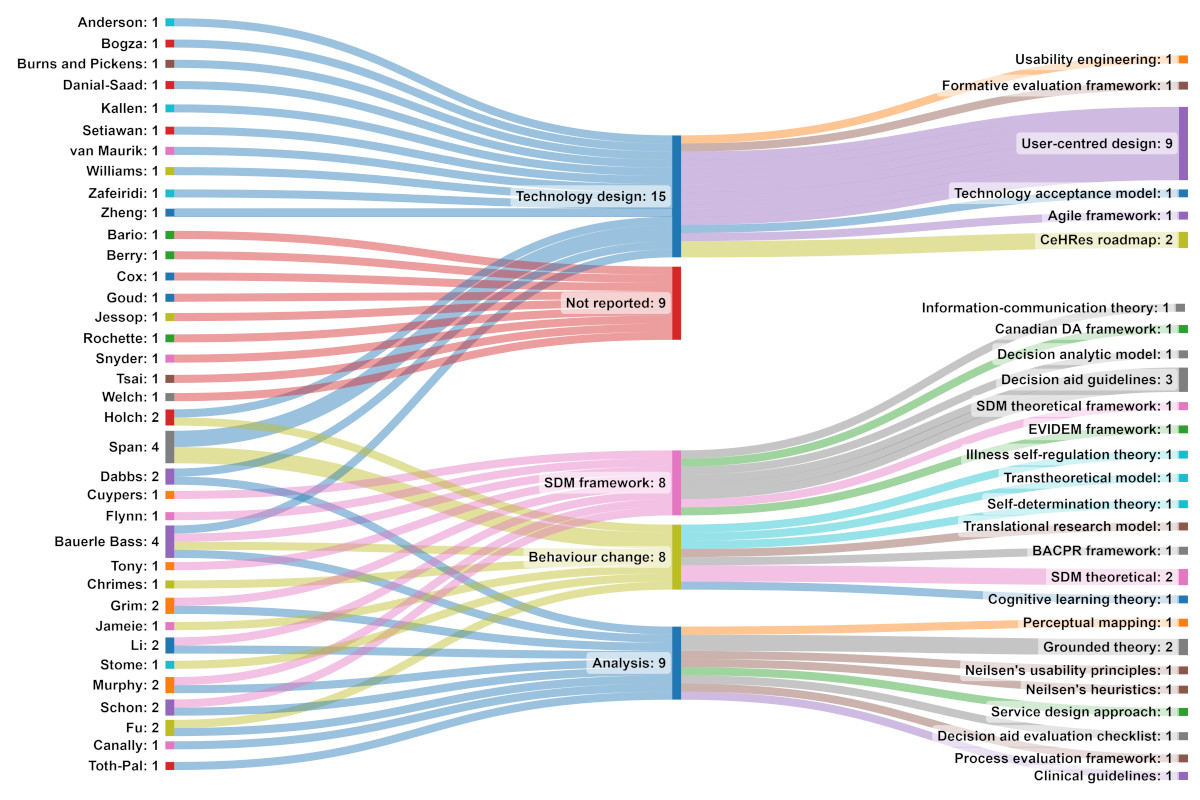
Mapping frameworks and theoretical models used during the usability evaluations. BACPR: British Association for Cardiovascular Prevention and Rehabilitation; CeHRes: Center for eHealth Research and Disease Management; DA: decision aid; EVIDEM: Evidence and Value: Impact on Decision-Making; SDM: shared decision-making. [32-41, 43, 45-69]

The frameworks and theoretical models reported by the authors during the development, implementation, and evaluation of the technologies to support SDM reflected 5 categories: technology design (15/38, 39%), behavior change (21/38, 21%), analysis (9/38, 24%), SDM framework (8/38, 21%), and not reported (9/38, 24%; Figure 4). Notably, 24% (9/38) of the studies did not report using a framework or model during any stage of their research. Authors most commonly reported using a model or framework as a foundation to inform the design of their respective SDM technologies. User-centered design (9/15, 60%) was the most frequently used technology design framework.

## Discussion

### Principal Findings

This scoping review was conducted to provide knowledge about how usability is evaluated when developing or implementing rehabilitation technologies aimed at supporting SDM. The first research question examined the methods and measures used in the context of SDM at different phases of technology development and implementation. Our findings revealed 14 reported methods that can help in evaluating the overall functionalities of the system and whether it fulfills the users’ requirements [75] and can be effective for identifying issues with a system [76]. The most frequent reported methods included think-aloud protocols (14/38, 36%), semistructured interviews (16/38, 42%), and questionnaires (24/38, 63%; Table 3). There was a total of 30 usability measures reported (Table 4), with the System Usability Scale being the most frequently used among the included studies. We operationalized the different types of methods used through the model by Jacobsen [31], reflecting empirical methods (based on users’ experience with the technology in a systematic way), inspection methods (conducted by experts who examine usability-related aspects of a user interface without involving any users), and inquiry methods (based on the information about users’ needs, likes, and understanding of the technology through interviews or focus groups, observation, or comments). Notably, the reported methods were predominantly classified as inquiry and empirical (Figure 2).

The second research question examined the parameters of usability that were measured and reported. We found that the methods used to evaluate different parameters of usability varied according to the a priori framing of usability, demonstrated by the variations in the definitions of usability described by the authors (Table 1). There was an evolution in the definition of usability across the included studies, with more recent studies (published since 2016) using the unified definition proposed by the ISO [43,46,48,57,61,64,65,67]. The usability parameters of the definitions were categorized based on the proposed comprehensive hierarchal model by Gupta et al [23] as effectiveness (9/38, 23%), efficiency (8/38, 21%), memorability (11/38, 29%), satisfaction (14/38, 37%), security (5/38, 13%), universality (4/38, 10%), and productivity (10/38, 26%). These are consistent with the 3 constructs of the ISO standards, which are effectiveness, efficiency, and satisfaction, and allows for a more detailed categorization of usability parameters.

Although the ISO standards [21] and the usability model by Gupta et al [23] provide dimensions that could be considered as primary usability parameters, there remain challenges with measuring usability that emerged in this review. On the surface, usability is a simple concept. In fact, simplicity is at the heart of usability; however, measuring usability is not simple. Paradoxically, the ISO definition of usability is complex. Usability is about the person’s experience; however, that experience is influenced by many aspects, such as a person’s behavior and social network and the complexity of the technological functionalities. Usability may be viewed as a feature of the technology or an emergent property of the interaction between the user, the system, and contextual factors. Evaluating usability through these lens leads to using inspection, empirical, or inquiry methods [31]. These can be applied at different stages of development of a technology (ie, in a developmental laboratory, in preimplementation, or during implementation), as described by the included studies (Table 2).

This review revealed that evaluating usability requires a comprehensive approach with several methods to cover multiple usability parameters. Most articles included in this review (36/38, 95%) focused on inquiry methods, relying heavily on questionnaires and semistructured interviews to evaluate usability, and the most frequent empirical method was think-aloud protocols (Figure 2). Although a comprehensive approach is suggested for accurate usability evaluation, this was largely not shown in the included articles. Rather, 73% (28/38) of the included studies only used 1 or 2 methods in total to evaluate usability. Only 2% (1/38) of the studies, conducted by Span et al [59], incorporated multiple methods that covered all 3 dimensions—inquiry, inspection, and empirical [31]. However, some of the included studies (2/38, 5%) described different usability evaluations for the same technology at different stages of development in separate articles (eg, “Take Charge, Get Cured” in the developmental [34] and preimplementation [51] stages). It is believed that the combination of inspection, empirical, and inquiry methods can provide more accurate and complete results in finding usability problems as there is no exact method considered to be the best for usability evaluation [77]. Matera et al [78] developed a systematic usability evaluation framework to address this challenge. They posited that usability can be reliably evaluated by systematically combining evaluation methods [78]. Recent reviews of usability not specific to SDM in software [79], mobile health [80], eHealth [81], user experience [82], and web development [83] mirrored the results of this review in that few studies used a combination of evaluation methods.

However, the lack of reported inspection methods demonstrated in this review may partially be explained by the inherent nature of SDM technologies for rehabilitation rather than a lack of comprehensive evaluation. Very few examples of inspection methods were demonstrated across the included studies, with only 2% (1/38) using cognitive walk-throughs and an additional 2% (1/38) using “near live” clinical situations. Critically, inspection methods refer to evaluations conducted by specific usability experts [31], not by the end users of the technology (eg, patients and clinicians). As the purpose of technology to support SDM in rehabilitation is to improve patient-centered care, the consideration of end users in the development—and, consequently, the usability evaluations—is crucial to ensure that the technology will be understood and adopted by the target population. Therefore, we propose that a comprehensive approach for evaluating the usability of rehabilitation technologies aimed at supporting SDM could focus on empirical and inquiry methods to prioritize the input of the patient and clinician end users.

Although questionnaires were found to be the most common method used overall, the identified measures of usability in the included studies demonstrated limitations in comprehensiveness, largely mapping to the parameters of satisfaction and memorability (Figure 3). The emphasis on the parameter of satisfaction (demonstrated in 32/38, 84% of measures) may reflect the importance of this parameter when developing technologies for SDM in rehabilitation (eg, the importance of evaluating the usefulness, user-friendliness, and ease of use). However, this may also reflect key missing areas in usability evaluation. Critically, the parameters of usability described by the authors in their a priori definitions of usability were not found to be consistent with the parameters of the measures that were used. Therefore, although individuals may be conceptualizing usability in a comprehensive manner, the measurement itself was not comprehensive. For example, there was a demonstrated lack of measurement of the parameters of effectiveness and efficiency, which were both described in the definition of usability in 34% (13/38) of the included studies, although both were only found to be used in 23% (9/38) of usability measures.

This review uncovered the need for inclusion of theoretical models or frameworks during various stages of SDM usability studies to guide which usability parameter to measure. Theoretical models and frameworks were infrequently reported (Figure 4). Most studies in this review (27/38, 71%) reported using 1 model or framework, whereas some (10/38, 26%) integrated 2. Only 2% (1/38) of the studies, carried out by Bauerle Bass et al [34], exhibited an in-depth application of models and frameworks as underpinnings to their research. The most common (9/38, 24%) and perhaps the most beneficial framework, user-centered design, served as the foundation for designing an SDM technology [21,36,42].

The importance of using theoretical models and frameworks during the development, implementation, and analysis of technologies and evaluation of usability is demonstrated through the implications of poor usability [18,84,85], which discourages users from using the technology systems. Moreover, if the technology systems are not user-friendly, then they can increase the problems experienced by users. Solutions to systems failing to meet the users’ needs include understanding user feedback [86], usability evaluations [75], involving users in the early stages of development [87], and including professionals such as providers [88]. There is a need for flexibility and for friendly, simple, and self-explanatory interfaces that allow users to interact with the system [89]. For the systems to be effective, it is important to assess a system that is easy to use on a daily basis. This would increase the ability of the patients to control their diseases and allow their daily lives to be more satisfying [76]. The technology systems need to be designed for a particular type of user and need to be easy to use to create acceptance. The usability of the technology system is vital as it has a high degree of influence over the success of the system. Thus, the system needs to be designed to provide a friendly environment for the user to develop a positive attitude toward using it and lead to its successful adoption.

It is envisioned that the involvement of end users in the development of SDM technologies will continue to grow and that more applications of existing technology, such as mobile phones, websites, or applications, will be used to benefit individuals with disabilities. We also anticipate that more companies may show an interest in this market, potentially promoting frequent use of SDM technologies in rehabilitation care. However, there are challenges in the development of SDM technologies, such as tailoring to individuals’ capabilities and properly addressing the emotional state of individuals with disabilities or cognitive impairments during everyday tasks. It will be critical to develop these technologies in a way that meets individual variations in needs and abilities of individuals with disabilities so that they really help maintain autonomy, provide meaningful activities, and promote decision-making [18,84,85].

An important area for this growing field will be how to effectively integrate end-user input throughout all stages of development of such SDM technologies, including effective usability testing. An additional challenge for the field of rehabilitation care in supporting SDM technologies would be in integrating the technology into the built environment, such as a client-server system, and into routine care [86]. There is a clear need for new methods of rapid SDM technology appraisal and evaluation to inform deployment to overcome the barriers that will be faced because of the expected further integration of SDM technologies within the built environment.

### Limitations

We did not assess the quality of the included articles, consistent with the scoping review methodology [27,90]. Therefore, we included studies with different designs and different quality levels, which allowed for a broad exploration of measures and methods used to evaluate the usability of SDM technologies. In our results, we focused mainly on general usability measures and did not report the psychometric properties and clinical utility of these measures. Future work needs to evaluate the psychometric properties and clinical utility of usability measures through a systematic review methodology with a quality assessment of the included articles. Another limitation was that we did not include gray literature as this scoping review aimed to examine the reported measures and methods used in peer-reviewed rehabilitation literature on SDM technologies. It could be an area of interest for future work to examine what methods and measures are used in gray literature.

### Conclusions

The results of this scoping review highlight the importance as well as the complexity of usability evaluation. Although various methods and measures were shown to be used to evaluate the usability of technologies to support SDM in rehabilitation, very few evaluations used in the included studies adequately spanned the selected usability parameters. This review identified gaps in usability evaluation as most studies relied solely on questionnaires rather than a combination of inspection and empirical methods and most questionnaires simply focused on the usability parameter of satisfaction. We recommend for individuals to adopt a comprehensive approach to usability evaluation of SDM technologies, starting with a clear definition of how usability is conceptualized to guide the structure of the evaluation. In addition, we recommend the use of multiple usability evaluation methods categorized as inspection (eg, questionnaires, focus groups, and interviews) or empirical (eg, think-aloud protocols) to capture a more complete picture of end-user needs and interpretations. The selected methods should span a variety of parameters of usability, not just satisfaction (eg, effectiveness, efficiency, memorability, security, universality, and productivity). The consideration of end users (such as patients and clinicians) is of particular importance for the development of technologies to support SDM as the process of SDM itself aims to improve patient-centered care and integrate both patient and clinician voices into their rehabilitation care.

## Appendix

Multimedia Appendix 1 Ovid MEDLINE search strategy.

Multimedia Appendix 2 Characteristics of the included studies and participants.

## Abbreviations

ISO: International Organization for Standardization
PRISMA-ScR: Preferred Reporting Items for Systematic Reviews and Meta-Analyses extension for Scoping Reviews
SDM: shared decision-making

## References

[1] Barry MJ, Edgman-Levitan S (2012). Shared decision making--pinnacle of patient-centered care. N Engl J Med.

[2] Sandman L, Munthe C (2010). Shared decision making, paternalism and patient choice. Health Care Anal.

[3] Légaré F, Witteman HO (2013). Shared decision making: examining key elements and barriers to adoption into routine clinical practice. Health Aff (Millwood).

[4] Davis S, MacKay L (2020). Moving beyond the rhetoric of shared decision-making: designing personal health record technology with young adults with type 1 diabetes. Can J Diabetes.

[5] Zisman-Ilani Y, Gorbenko KO, Shern D, Elwyn G (2017). Comparing digital vs paper decision aids about the use of antipsychotic medication: client, clinician, caregiver and administrator perspectives. Int J Pers Cent Med.

[6] Rose A, Rosewilliam S, Soundy A (2017). Shared decision making within goal setting in rehabilitation settings: a systematic review. Patient Educ Couns.

[7] Grenfell J, Soundy A (2022). People's Experience of Shared Decision Making in Musculoskeletal Physiotherapy: A Systematic Review and Thematic Synthesis. Behav Sci (Basel).

[8] Matthews EB, Savoy M, Paranjape A, Washington D, Hackney T, Galis D, Zisman-Ilani Y (2022). Acceptability of health information exchange and patient portal use in depression care among underrepresented patients. J Gen Intern Med.

[9] Zisman-Ilani Y, Roe D, Elwyn G, Kupermintz H, Patya N, Peleg I, Karnieli-Miller O (2019). Shared decision making for psychiatric rehabilitation services before discharge from psychiatric hospitals. Health Commun.

[10] Shay LA, Lafata JE (2015). Where is the evidence? A systematic review of shared decision making and patient outcomes. Med Decis Making.

[11] Wilson SR, Strub P, Buist AS, Knowles SB, Lavori PW, Lapidus J, Vollmer WM, Better Outcomes of Asthma Treatment (BOAT) Study Group (2010). Shared treatment decision making improves adherence and outcomes in poorly controlled asthma. Am J Respir Crit Care Med.

[12] Hartasanchez SA, Heen AF, Kunneman M, García-Bautista A, Hargraves IG, Prokop LJ, May CR, Montori VM (2022). Remote shared decision making through telemedicine: a systematic review of the literature. Patient Educ Couns.

[13] Safran Naimark J, Madar Z, Shahar DR (2015). The impact of a web-based app (eBalance) in promoting healthy lifestyles: randomized controlled trial. J Med Internet Res.

[14] Solomon M, Wagner SL, Goes J (2012). Effects of a web-based intervention for adults with chronic conditions on patient activation: online randomized controlled trial. J Med Internet Res.

[15] Antonio MG, Petrovskaya O, Lau F (2020). The state of evidence in patient portals: umbrella review. J Med Internet Res.

[16] Seljelid B, Varsi C, Solberg Nes L, Øystese KA, Børøsund E (2022). Feasibility of a digital patient-provider communication intervention to support shared decision-making in chronic health care, involveme: pilot study. JMIR Form Res.

[17] Davis S, Roudsari A, Raworth R, Courtney KL, MacKay L (2017). Shared decision-making using personal health record technology: a scoping review at the crossroads. J Am Med Inform Assoc.

[18] Kao H, Wei C, Yu M, Liang T, Wu W, Wu YJ (2018). Integrating a mobile health applications for self-management to enhance telecare system. Telemat Inform.

[19] Taylor L, Capling H, Portnoy JM (2018). Administering a telemedicine program. Curr Allergy Asthma Rep.

[20] Howe TL, Worrall LE, Hickson LM (2010). What is an aphasia-friendly environment?. Aphasiology.

[21] Jokela T, Iivari N, Matero J, Karukka M (2003). The standard of user-centered design and the standard definition of usability: analyzing ISO 13407 against ISO 9241-11. Proceedings of the Latin American Conference on Human-Computer Interaction.

[22] Holzinger A (2005). Usability engineering methods for software developers. Commun ACM.

[23] Gupta D, Ahlawat AK, Sagar K (2017). Usability prediction and ranking of SDLC models using fuzzy hierarchical usability model. Open Eng.

[24] Schultheis MT, Rebimbas J, Mourant R, Millis SR (2007). Examining the usability of a virtual reality driving simulator. Assist Technol.

[25] Negrini S, Meyer T, Arienti C, Kiekens C, Pollock A, Selb M, Stucki G, 3rd Cochrane Rehabilitation Methodology Meeting participants (2020). The 3rd Cochrane rehabilitation methodology meeting: "rehabilitation definition for scientific research purposes". Eur J Phys Rehabil Med.

[26] Damman OC, Jani A, de Jong BA, Becker A, Metz MJ, de Bruijne MC, Timmermans DR, Cornel MC, Ubbink DT, van der Steen M, Gray M, van El C (2020). The use of PROMs and shared decision-making in medical encounters with patients: an opportunity to deliver value-based health care to patients. J Eval Clin Pract.

[27] Arksey H, O'Malley L (2005). Scoping studies: towards a methodological framework. Int J Soc Res Methodol.

[28] Tricco AC, Lillie E, Zarin W, O'Brien KK, Colquhoun H, Levac D, Moher D, Peters MD, Horsley T, Weeks L, Hempel S, Akl EA, Chang C, McGowan J, Stewart L, Hartling L, Aldcroft A, Wilson MG, Garritty C, Lewin S, Godfrey CM, Macdonald MT, Langlois EV, Soares-Weiser K, Moriarty J, Clifford T, Tunçalp Ö, Straus SE (2018). PRISMA extension for scoping reviews (PRISMA-ScR): checklist and explanation. Ann Intern Med.

[29] Gammon D, Berntsen GK, Koricho AT, Sygna K, Ruland C (2015). The chronic care model and technological research and innovation: a scoping review at the crossroads. J Med Internet Res.

[30] Higgins J, Green S (2008). Cochrane Handbook for Systematic Reviews of Interventions.

[31] Jacobsen NE (1999). Usability evaluation methods: the reliability and usage of cognitive walkthrough and usability test. Department of Psychology, University of Copenhagen.

[32] Anderson JA, Godwin KM, Saleem JJ, Russell S, Robinson JJ, Kimmel B (2014). Accessibility, usability, and usefulness of a web-based clinical decision support tool to enhance provider-patient communication around self-management TO prevent (STOP) stroke. Health Informatics J.

[33] Barrio P, Ortega L, López H, Gual A (2017). Self-management and shared decision-making in alcohol dependence via a mobile app: a pilot study. Int J Behav Med.

[34] Bauerle Bass S, Jessop A, Gashat M, Maurer L, Alhajji M, Forry J (2018). Take charge, get cured: the development and user testing of a culturally targeted mHealth decision tool on HCV treatment initiation for methadone patients. Patient Educ Couns.

[35] Berry DL, Halpenny B, Bosco JL, Bruyere Jr J, Sanda MG (2015). Usability evaluation and adaptation of the e-health personal patient profile-prostate decision aid for Spanish-speaking Latino men. BMC Med Inform Decis Mak.

[36] Bogza LM, Patry-Lebeau C, Farmanova E, Witteman HO, Elliott J, Stolee P, Hudon C, Giguere AM (2020). User-centered design and evaluation of a web-based decision aid for older adults living with mild cognitive impairment and their health care providers: mixed methods study. J Med Internet Res.

[37] Burns SP, Pickens ND (2017). Embedding technology into inter-professional best practices in home safety evaluation. Disabil Rehabil Assist Technol.

[38] Canally C, Doherty S, Doran DM, Goubran RA (2015). Using integrated bio-physiotherapy informatics in home health-care settings: a qualitative analysis of a point-of-care decision support system. Health Informatics J.

[39] Chrimes D, Kitos NR, Kushniruk A, Mann DM (2014). Usability testing of avoiding diabetes thru action plan targeting (ADAPT) decision support for integrating care-based counseling of pre-diabetes in an electronic health record. Int J Med Inform.

[40] Cox CE, Wysham NG, Walton B, Jones D, Cass B, Tobin M, Jonsson M, Kahn JM, White DB, Hough CL, Lewis CL, Carson SS (2015). Development and usability testing of a web-based decision aid for families of patients receiving prolonged mechanical ventilation. Ann Intensive Care.

[41] Cuypers M, Lamers RE, Kil PJ, The R, Karssen K, van de Poll-Franse LV, de Vries M (2019). A global, incremental development method for a web-based prostate cancer treatment decision aid and usability testing in a Dutch clinical setting. Health Informatics J.

[42] De Vito Dabbs A, Myers BA, Mc Curry KR, Dunbar-Jacob J, Hawkins RP, Begey A, Dew MA (2009). User-centered design and interactive health technologies for patients. Comput Inform Nurs.

[43] Danial-Saad A, Kuflik T, Weiss PL, Schreuer N (2016). Usability of clinical decision support system as a facilitator for learning the assistive technology adaptation process. Disabil Rehabil Assist Technol.

[44] Fleisher L, Buzaglo J, Collins M, Millard J, Miller SM, Egleston BL, Solarino N, Trinastic J, Cegala DJ, Benson 3rd AB, Schulman KA, Weinfurt KP, Sulmasy D, Diefenbach MA, Meropol NJ (2008). Using health communication best practices to develop a web-based provider-patient communication aid: the CONNECT study. Patient Educ Couns.

[45] Flynn AJ, Friedman CP, Boisvert P, Landis-Lewis Z, Lagoze C (2018). The knowledge object reference ontology (KORO): a formalism to support management and sharing of computable biomedical knowledge for learning health systems. Learn Health Syst.

[46] Fu H, Rizvi RF, Wyman JF, Adam TJ (2020). Usability evaluation of four top-rated commercially available diabetes apps for adults with type 2 diabetes. Comput Inform Nurs.

[47] Goud R, Jaspers MW, Hasman A, Peek N (2008). Subjective usability of the CARDSS guideline-based decision support system. Stud Health Technol Inform.

[48] Grim K, Rosenberg D, Svedberg P, Schön UK (2017). Development and usability testing of a web-based decision support for users and health professionals in psychiatric services. Psychiatr Rehabil J.

[49] Holch P, Warrington L, Bamforth LC, Keding A, Ziegler LE, Absolom K, Hector C, Harley C, Johnson O, Hall G, Morris C, Velikova G (2017). Development of an integrated electronic platform for patient self-report and management of adverse events during cancer treatment. Ann Oncol.

[50] Jameie S, Haybar H, Aslani A, Saadat M (2019). Development and usability evaluation of web-based telerehabilitation platform for patients after myocardial infarction. Stud Health Technol Inform.

[51] Jessop AB, Bass SB, Brajuha J, Alhajji M, Burke M, Gashat MT, Wellington C, Ventriglia N, Coleman J, D'Avanzo P (2020). "Take charge, get cured": pilot testing a targeted mHealth treatment decision support tool for methadone patients with hepatitis C virus for acceptability and promise of efficacy. J Subst Abuse Treat.

[52] Kallen MA, Yang D, Haas N (2012). A technical solution to improving palliative and hospice care. Support Care Cancer.

[53] Li LC, Adam PM, Townsend AF, Lacaille D, Yousefi C, Stacey D, Gromala D, Shaw CD, Tugwell P, Backman CL (2013). Usability testing of ANSWER: a web-based methotrexate decision aid for patients with rheumatoid arthritis. BMC Med Inform Decis Mak.

[54] Murphy C, de Laine C, Macaulay M, Fader M (2020). Development and randomised controlled trial of a continence product patient decision aid for men postradical prostatectomy. J Clin Nurs.

[55] Rochette A, Korner-Bitensky N, Tremblay V, Kloda L (2008). Stroke rehabilitation information for clients and families: assessing the quality of the strokengine-family website. Disabil Rehabil.

[56] Schön UK, Grim K, Wallin L, Rosenberg D, Svedberg P (2018). Psychiatric service staff perceptions of implementing a shared decision-making tool: a process evaluation study. Int J Qual Stud Health Well-being.

[57] Setiawan IM, Zhou L, Alfikri Z, Saptono A, Fairman AD, Dicianno BE, Parmanto B (2019). An adaptive mobile health system to support self-management for persons with chronic conditions and disabilities: usability and feasibility studies. JMIR Form Res.

[58] Snyder CF, Jensen R, Courtin SO, Wu AW, Website for Outpatient QOL Assessment Research Network (2009). PatientViewpoint: a website for patient-reported outcomes assessment. Qual Life Res.

[59] Span M, Hettinga M, Groen-van de Ven L, Jukema J, Janssen R, Vernooij-Dassen M, Eefsting J, Smits C (2018). Involving people with dementia in developing an interactive web tool for shared decision-making: experiences with a participatory design approach. Disabil Rehabil.

[60] Span M, Smits C, Jukema J, Groen-van de Ven L, Janssen R, Vernooij-Dassen M, Eefsting J, Hettinga M (2014). An interactive web tool to facilitate shared decision-making in dementia: design issues perceived by caregivers and patients. Int J Adv Life Sci.

[61] Støme LN, Pripp AH, Kværner JS, Kvaerner KJ (2019). Acceptability, usability and utility of a personalised application in promoting behavioural change in patients with osteoarthritis: a feasibility study in Norway. BMJ Open.

[62] Tony M, Wagner M, Khoury H, Rindress D, Papastavros T, Oh P, Goetghebeur MM (2011). Bridging health technology assessment (HTA) with multicriteria decision analyses (MCDA): field testing of the EVIDEM framework for coverage decisions by a public payer in Canada. BMC Health Serv Res.

[63] Toth-Pal E, Wårdh I, Strender LE, Nilsson G (2008). Implementing a clinical decision-support system in practice: a qualitative analysis of influencing attitudes and characteristics among general practitioners. Inform Health Soc Care.

[64] Tsai YL, Huang JJ, Pu SW, Chen HP, Hsu SC, Chang JY, Pei YC (2019). Usability assessment of a cable-driven exoskeletal robot for hand rehabilitation. Front Neurorobot.

[65] van Maurik IS, Visser LN, Pel-Littel RE, van Buchem MM, Zwan MD, Kunneman M, Pelkmans W, Bouwman FH, Minkman M, Schoonenboom N, Scheltens P, Smets EM, van der Flier WM (2019). Development and usability of ADappt: web-based tool to support clinicians, patients, and caregivers in the diagnosis of mild cognitive impairment and Alzheimer disease. JMIR Form Res.

[66] Welch G, Balder A, Zagarins S (2015). Telehealth program for type 2 diabetes: usability, satisfaction, and clinical usefulness in an urban community health center. Telemed J E Health.

[67] Williams PA, Furberg RD, Bagwell JE, LaBresh KA (2016). Usability testing and adaptation of the pediatric cardiovascular risk reduction clinical decision support tool. JMIR Hum Factors.

[68] Zafeiridi P, Paulson K, Dunn R, Wolverson E, White C, Thorpe JA, Antomarini M, Cesaroni F, Scocchera F, Landrin-Dutot I, Malherbe L, Lingiah H, Bérard M, Gironès X, Quintana M, Cortés U, Barrué C, Cortés A, Paliokas I, Votis K, Tzovaras D (2018). A web-based platform for people with memory problems and their caregivers (CAREGIVERSPRO-MMD): mixed-methods evaluation of usability. JMIR Form Res.

[69] Zheng H, Tulu B, Choi W, Franklin P (2017). Using mHealth app to support treatment decision-making for knee arthritis: patient perspective. EGEMS (Wash DC).

[70] Feldman-Stewart D, Brundage MD, Nickel JC, MacKillop WJ (2001). The information required by patients with early-stage prostate cancer in choosing their treatment. BJU Int.

[71] Feldman-Stewart D, Brundage MD, Van Manen L, Svenson O (2004). Patient-focussed decision-making in early-stage prostate cancer: insights from a cognitively based decision aid. Health Expect.

[72] Feldman-Stewart D, Brennenstuhl S, Brundage MD, Roques T (2006). An explicit values clarification task: development and validation. Patient Educ Couns.

[73] Feldman-Stewart D, Brundage MD, Zotov V (2007). Further insight into the perception of quantitative information: judgments of gist in treatment decisions. Med Decis Making.

[74] Feldman-Stewart D, Tong C, Siemens R, Alibhai S, Pickles T, Robinson J, Brundage MD (2012). The impact of explicit values clarification exercises in a patient decision aid emerges after the decision is actually made: evidence from a randomized controlled trial. Med Decis Making.

[75] van Osch M, Rövekamp A, Bergman-Agteres SN, Wijsman LW, Ooms SJ, Mooijaart SP, Vermeulen J (2015). User preferences and usability of iVitality: optimizing an innovative online research platform for home-based health monitoring. Patient Prefer Adherence.

[76] Lilholt PH, Jensen MH, Hejlesen OK (2015). Heuristic evaluation of a telehealth system from the Danish TeleCare north trial. Int J Med Inform.

[77] Paz F, Paz FA, Villanueva D, Pow-Sang JA (2015). Heuristic evaluation as a complement to usability testing: a case study in web domain. Proceedings of the 12th International Conference on Information Technology-New Generations.

[78] Matera M, Costabile MF, Garzotto F, Paolini P (2002). SUE inspection: an effective method for systematic usability evaluation of hypermedia. IEEE Transactions on Systems, Man, and Cybernetics-Part A: Systems and Humans.

[79] Sagar K, Saha A (2017). A systematic review of software usability studies. Int J Inf Technol.

[80] Al-Saadi TA, Aljarrah TM, Alhashemi AM, Hussain A (2015). A systematic review of usability challenges and testing in mobile health. Int J Account Financ Report.

[81] Maramba I, Chatterjee A, Newman C (2019). Methods of usability testing in the development of eHealth applications: a scoping review. Int J Med Inform.

[82] Maia CL, Furtado ES (2016). A systematic review about user experience evaluation. Proceedings of the 5th International Conference on Design, User Experience, and Usability: Design Thinking and Methods.

[83] Insfran E, Fernandez A (2008). A systematic review of usability evaluation in web development. Proceedings of the 2008 International Workshops on Web Information Systems Engineering.

[84] Cook EJ, Randhawa G, Guppy A, Sharp C, Barton G, Bateman A, Crawford-White J (2018). Exploring factors that impact the decision to use assistive telecare: perspectives of family care-givers of older people in the United Kingdom. Ageing Soc.

[85] Martínez García MA, Fernández Rosales MS, López Domínguez E, Hernández Velázquez Y, Domínguez Isidro S (2018). Telemonitoring system for patients with chronic kidney disease undergoing peritoneal dialysis: usability assessment based on a case study. PLoS One.

[86] Saeed N, Manzoor M, Khosravi P (2020). An exploration of usability issues in telecare monitoring systems and possible solutions: a systematic literature review. Disabil Rehabil Assist Technol.

[87] Sánchez-Morillo D, Crespo M, León A, Crespo Foix LF (2015). A novel multimodal tool for telemonitoring patients with COPD. Inform Health Soc Care.

[88] Klack L, Ziefle M, Wilkowska W, Kluge J (2013). Telemedical versus conventional heart patient monitoring: a survey study with German physicians. Int J Technol Assess Health Care.

[89] Prescher S, Bourke AK, Koehler F, Martins A, Ferreira HS, Sousa TB, Castro RN, Santos A, Torrent M, Gomis S, Hospedales M, Nelson J (2012). Ubiquitous ambient assisted living solution to promote safer independent living in older adults suffering from co-morbidity. Proceedings of the 2012 Annual International Conference of the IEEE Engineering in Medicine and Biology Society.

[90] Daudt HM, van Mossel C, Scott SJ (2013). Enhancing the scoping study methodology: a large, inter-professional team's experience with Arksey and O'Malley's framework. BMC Med Res Methodol.

