# Supplementary material for: Methodologies for Evaluating the Usability of Rehabilitation Technologies Aimed at Supporting Shared Decision-Making: Scoping Review
Source: JMIR Rehabil Assist Technol. 2023 Aug 15;10:e41359. doi: 10.2196/41359 (PMC10466154; doi:10.2196/41359)
Supplement: Multimedia Appendix 1 [file rehab_v10i1e41359_app1.docx]

**Appendix 1. Medline Ovid search strategy**

1. (usability or "user* friendl*" or "eas* to use" or "eas* of use" or useful* or user* perspective* or patient* perspective* or client* perspective* or user* experience* or patient* experience* or client* experience* or overall impression* or general impression* or expectanc* or acceptanc* or acceptabilit*).ab,kf,kw,ti.

2. (user* adj2 interface*).ab,kf,kw,ti.

3. 1 or 2

4. exp Rehabilitation/

5. exp Rehabilitation Centers/

6. exp Disabled Persons/

7. exp Occupational Therapy/

8. exp Occupational Therapists/

9. exp Physical Therapy Modalities/ or exp Physical Therapy Specialty/

10. exp Physical Therapists/

11. exp Speech-Language Pathology/

12. exp Communication Disorders/

13. exp Vision Disorders/

14. exp Cognition Disorders/

15. exp Visually Impaired Persons/

16. (rehabilitation or telerehabilitation or tele rehabilitation or erehabilitation or e-rehabilitation or disabled or disabilit* or physical limitation* or mental limitation* or psycho* limitation* or adaptation* or mobility or occupational therap* or physiotherap* or physical therap* or speech languag* pathol* or speech therap* or language therap* or communication disorder* or visual impair* or visual* disorder* or vision impair* or impair* vision or vision disorder* or cognit* impair* or cognit* disorder* or blindness).ab,kf,kw,ti.

17. rehabilitation.fs,fx,xs.

18. 4 or 5 or 6 or 7 or 8 or 9 or 10 or 11 or 12 or 13 or 14 or 15 or 16 or 17

19. exp "Surveys and Questionnaires"/

20. exp Focus Groups/

21. exp INTERVIEW/

22. exp Psychometrics/

23. ("think* aloud" or "focus group*" or interview* or Wizard* or "Empathy map*" or Persona* or Questionnaire* or instrument* or scale* or tool or tools or measurement* or survey* or drama or deliberation* or evaluation* or assessment* or video confrontation* or photo voice*).ab,kf,kw,ti.

24. 19 or 20 or 21 or 22 or 23

25. exp Telemedicine/

26. exp Technology/

27. exp Biosensing Techniques/

28. exp Remote Sensing Technology/

29. exp Self-Help Devices/

30. exp Monitoring, Ambulatory/

31. exp Smartphone/

32. exp Mobile Applications/

33. exp Virtual Reality/

34. exp Computers/

35. exp Computer Simulation/

36. exp SOFTWARE/

37. exp Communication Aids for Disabled/

38. exp Wheelchairs/

39. exp Geographic Information Systems/

40. exp INTERNET/

41. (technolog* or gerontotechnolog* or smart* or intelligen* or ambient assisted living or virtual reality or virtual rehabilitation or telemonitoring or telehealth or telemedicine or telerehabilitation or ehealth or tele monitoring or tele health or tele medicine or tele rehabilitation or e health or sensor* or biosensor* or mobile app* or product* or internet or web or computer* or software* or device* or self-help or wheelchair* or wheel chair* or communication aid* or augmentative communication* or alternative communication* or electronic or detector* or geolocalisation or geolocalization).ab,kf,kw,ti.
42. shared decision making,” “Decision Making” [MeSH], “patient-provider communication,” “decision aid,” “decision support”; and we can add more such as: Clinical decision-support systems, clinical interaction and support for self-management.

43. 25 or 26 or 27 or 28 or 29 or 30 or 31 or 32 or 33 or 34 or 35 or 36 or 37 or 38 or 39 or 40 or

44. 3 and 18 and 24 and 42
45. limit 44 to yr="2005 -Current"
46. exp Models, Animal/
47. exp MICE/
48. 46 or 47
49. 46 not 47
50. limit 48 to (comment or editorial)
51. 48 not 49
52. limit 44 to (english or french)
