# Supplementary material for: Methodologies for Evaluating the Usability of Rehabilitation Technologies Aimed at Supporting Shared Decision-Making: Scoping Review
Source: JMIR Rehabil Assist Technol. 2023 Aug 15;10:e41359. doi: 10.2196/41359 (PMC10466154; doi:10.2196/41359)
Supplement: Multimedia Appendix 2 [file rehab_v10i1e41359_app2.docx]

**Appendix 2.** Characteristics of the included studies and participants.

| Study | Country | Study design | Recruitment setting | Type of population—patients or caregivers | Type of population—providers | Target user of technology | Total number of participants, N | Age (years), mean±SD or (range) |
| --- | --- | --- | --- | --- | --- | --- | --- | --- |
| Anderson et al [32], 2014 | United States | Qualitative | Stroke Quality Improvement Network, Michael E. DeBakey Veterans Affairs Medical Center, and Richard L. Roudebush Veterans’ Administration Medical Center (Hospital) | Individuals with ischemic stroke or transient ischemic attack | Stroke Quality Improvement Network evaluators and multidisciplinary stroke care providers | Clinicians and patients | 22 | NR^a^ |
| Barrio et al [33], 2017 | Spain | Qualitative | Outpatient service of Additions Unit at Hospital Clinic of Barcelona (Hospital) | Individuals with alcohol dependence | Psychiatrist therapists | Clinicians and patients | 29 | 48±11.3 |
| Bauerle Bass et al [34], 2018 | United States | Mixed methods | 4 Philadelphia methadone programs (Hospital) | Patients with hepatitis C taking methadone | Physician (hepatitis C specialist) | Patients | 10 | 42 (33-50) |
| Berry et al [35], 2015 | United States | Qualitative | Genitourinary oncology clinic and urology clinics of academic medical centers (Hospital) | Individuals with a localized prostate cancer | Clinicians | Clinicians and patients | 7 | (54-67) |
| Bogza et al [36], 2020 | Canada | Mixed methods | Primary care clinics  (Hospital) | Individuals with mild cognitive impairment | Health care providers | Clinicians and patients | 12 | (60-86) |
| Burns and Pickens [37], 2017 | United States | Qualitative | Clinics from 7 regions of the United States  (Hospital) | Adults with disabilities | Home evaluators (expert/novice occupational therapists, occupational therapy assistant, physiotherapists, design professionals, contractor, and social service professional) | Home evaluators | 20 | NR |
| Canally et al [38], 2015 | Canada | Qualitative | Home care service agency  (Hospital) | Older adults who lived independently in their home | Home care professionals (home care nurses, physiotherapists, occupational therapists, and case managers) | Home care professionals | 8 | NR |
| Chrimes et al [39], 2014 | United States | Qualitative | General medicine clinic associated with the hosting academic institution  (Hospital) | Individuals with prediabetes | Primary care providers | Clinicians and patients | 13 | NR |
| Cox et al [40], 2015 | United States | Mixed methods | ICU^b^ waiting room  (Hospital) | Critical illness survivors who received mechanical ventilation and family members of patients in the ICU | Surrogate decision makers of ICU survivors | Surrogate decision makers of ICU survivors | 108 | (45-66) |
| Cuypers et al [41], 2019 | The Netherlands | Mixed methods | Oncology clinic in Southern Netherlands  (Hospital) | Individuals with cancer | Oncologists, nurses, and urologists | Clinicians and patients | 11 | (55-65) |
| De Vito Dabbs et al [42], 2009 | United States | Mixed methods | Pulmonary transplant center  (Hospital) | Individuals with lung transplant | Nurse, computer scientist, behavioral scientist, cardiothoracic transplant physician, and communication scientist | Clinicians and patients | 7 | (21-69) |
| Danial-Saad et al [43], 2016 | Israel | Quantitative | NR | Individuals requiring assistive technology adaptations | Occupational therapists | Clinicians | 26 | (26-51) |
| Fleisher et al [44], 2008 | United States | Mixed methods | Fox Chase Cancer Center  (Hospital) | Individuals with advanced cancer | Oncologists | Clinicians and patients | 888 | (37-77) |
| Flynn et al [45], 2015 | United Kingdom | Mixed methods | 3 acute stroke units  (Hospital) | Individuals with acute stroke and caregivers | Stroke physicians, emergency department physicians, and stroke nurse practitioners | Clinicians, patients, and caregivers | 22 | NR |
| Fu et al [46], 2020 | United States | Mixed methods | US hospital  (Hospital) | Individuals with diabetes | Health care providers | Clinicians and patients | NR | NR |
| Goud et al [47], 2008 | The Netherlands | Quantitative | Outpatient clinics  (Hospital) | Individuals with postmyocardial infarctions | Cardiac rehabilitation professionals, nurses, and paramedics | Cardiac rehabilitation professionals | 63 | 42.8±8.2 |
| Grim et al [48], 2017 | Sweden | Qualitative | Community-based mental health services  Community | Individuals with mental illnesses and caregivers | Service providers and supported housing workers | Clinicians, patients, and caregivers | 86 | (45-55) |
| Holch et al [49], 2017 | United Kingdom | Mixed methods | Leeds Cancer Centre  (Hospital) | Patients experiencing adverse effects during cancer therapy | Clinicians, clinical staff, and administrators | Clinicians and patients | 41 | (35-69) |
| Jameie et al [50], 2019 | Iran | Mixed methods | Cardiac rehabilitation  (Hospital) | Postmyocardial infarction | Health care providers and engineers | Clinicians and patients | 15 | NR |
| Jessop et al [51], 2020 | United States | Quantitative | Philadelphia methadone maintenance treatment programs  (Hospital) | Patients with hepatitis C taking methadone | Physician | Patients | 122 | NR |
| Kallen et al [52], 2012 | United States | Mixed methods | Department of Palliative Care and Rehabilitation Medicine at the University of Texas MD Anderson Cancer Center  University | Individuals with cancer and caregivers | Physicians and nurses | Clinicians and patients | 81 | NR |
| Li et al [53], 2013 | Canada | Mixed methods | Rheumatologist office, community health centers, Mary Pack Arthritis Program, Vancouver General Hospital, Craigslist, and Kijiji  Community | Individuals with rheumatoid arthritis | —^c^ | Patients | 15 | (20-≥65) |
| Murphy et al [54], 2020 | United Kingdom | Mixed methods | “Trial Without Catheter” clinics in 4 NHS^d^ acute trusts  (Hospital) | Men following radical prostatectomy | Clinicians who regularly assessed patients for continence products | Clinicians and patients | 21 | NR |
| Rochette et al [55], 2008 | Canada | Mixed methods | Telephone  Community | Individuals with stroke and family | —^c^ | Individuals with stroke and family | 7 | (43-68) |
| Schön et al [56], 2018 | Sweden | Mixed methods | Psychiatric units across Sweden  (Hospital) | Patients with severe mental illness | Health care providers | Clinicians and patients | 95 | 45 (24-65) |
| Setiawan et al [57], 2019 | United States | Mixed methods | General public of the Greater Pittsburgh area  Community | Individuals with chronic conditions and disabilities and caregivers | Clinicians | Clinicians and patients | 81 | 30.4±12.82 |
| Snyder et al [58], 2009 | United States | Qualitative | Sidney Kimmel Comprehensive Cancer Center at Johns Hopkins  (Hospital) | Individuals with cancer | Oncologists and research nurses | Clinicians and patients | 37 | NR |
| Span et al [60], 2014 | The Netherlands | Qualitative | Case manager network representing organizations providing dementia care  Community | Individuals with dementia and caregivers | Case managers | Patients, caregivers, and case managers | 19 | (19-62) |
| Span et al [59], 2018 | The Netherlands | Quantitative | Nursing homes, day-care settings, and website of the Dutch Alzheimer’s Association  Community | Individuals with mild to moderate dementia and caregivers | Case managers | Patients, caregivers, and case managers | 51 | (62-86) |
| Støme et al [61], 2019 | Norway | Mixed methods | Rheumatology clinic at Diakonhjemmet Hospital, Oslo, Norway  (Hospital) | Individuals with osteoarthritis | Health care providers | Clinicians and patients | 11 | 65 (52-79) |
| Tony et al [62], 2011 | Canada | Mixed methods | Ontario Workplace Safety and Insurance Board  Community | Workers experiencing injury or illness directly related to work | Drug advisory committee | Health care professionals and decision makers | 9 | NR |
| Toth-Pal et al [63], 2008 | Sweden | Qualitative | Primary health care center  (Hospital) | Individuals with chronic heart failure | General practitioners | Clinicians | 5 | NR |
| Tsai et al [64], 2019 | United States | Quantitative | Acute inpatient rehabilitation unit and community  (Hospital and Community) | Family members or designated person of patients requiring DME^e^ | Clinicians responsible for ordering DME (occupational therapist and physical therapists) | Clinicians and caregivers | 38 | (18-75) |
| Van Maurik et al [65], 2019 | The Netherlands | Mixed methods | Vrije Universiteit Amsterdam  (University) | Patients with cognitive impairment and Alzheimer disease | Health care providers | Clinicians and patients | 5 | 65±9 |
| Welch et al [66], 2015 | United States | Quantitative | Community health center of a medically underserved, urban neighborhood  (Community) | Individuals with poorly controlled type 2 diabetes | Clinicians | Clinicians and patients | 42 | 60.6±7.1 |
| Williams et al [67], 2016 | United States | Qualitative | Universities and American Academy of Pediatrics Quality Improvement Innovations Network  (University) | Children and adolescents at risk of cardiovascular disease | Pediatricians | Pediatricians | 34 | NR |
| Zafeiridi et al [68], 2018 | Italy, United Kingdom, Spain, and France | Mixed methods | Local health and social care providers and community support groups in Ancona, Hull, Manresa, and Rouen  (Community) | People with dementia or mild cognitive impairment and caregivers | Health and social care professionals | Clinicians, patients, and caregivers | 58 | (26-91) |
| Zheng et al [69], 2017 | United States | Qualitative | University of Massachusetts Memorial Arthritis and Joint Replacement Center  (University) | Individuals with advanced knee arthritis | Surgeons and physiotherapists | Clinicians and patients | 19 | (55-80) |

^a^NR: not reported.

^b^ICU: intensive care unit.

^c^Data not available.

^d^NHS: National Health Service.

^e^DME: durable medical equipment.
